# HGV&TB: a comprehensive online resource on human genes and genetic variants associated with tuberculosis

**DOI:** 10.1093/database/bau112

**Published:** 2014-12-13

**Authors:** Ruchika Sahajpal, Gaurav Kandoi, Heena Dhiman, Sweety Raj, Open Source Drug Discovery Consortium, Vinod Scaria, Deeksha Bhartiya, Yasha Hasija

**Affiliations:** ^1^ Department of Biotechnology, Delhi Technological University, Bawana Road, Delhi 110042, India, ^2^GN Ramachandran Knowledge Center for Genome Informatics, CSIR-Institute of Genomics and Integrative Biology (CSIR-IGIB), Mathura Road, Delhi 110025, India, ^3^Acharya Narendra Dev College, University of Delhi, Govindpuri, Kalkaji, New Delhi 110019, India, ^4^Council of Scientific and Industrial Research (CSIR), Anusandhan Bhawan, 2 Rafi Marg, New Delhi 110001, India and ^5^Academy of Scientific and Innovative Research (AcSIR), Anusandhan Bhawan, New Delhi 110001, India

## Abstract

Tuberculosis (TB) is an infectious disease caused by fastidious pathogen *Mycobacterium tuberculosis*. TB has emerged as one of the major causes of mortality in the developing world. Role of host genetic factors that modulate disease susceptibility have not been studied widely. Recent studies have reported few genetic loci that provide impetus to this area of research. The availability of tools has enabled genome-wide scans for disease susceptibility loci associated with infectious diseases. Till now, information on human genetic variations and their associated genes that modulate TB susceptibility have not been systematically compiled. In this work, we have created a resource: HGV&TB, which hosts genetic variations reported to be associated with TB susceptibility in humans. It currently houses information on 307 variations in 98 genes. In total, 101 of these variations are exonic, whereas 78 fall in intronic regions. We also analysed the pathogenicity of the genetic variations, their phenotypic consequences and ethnic origin. Using various computational analyses, 30 variations of the 101 exonic variations were predicted to be pathogenic. The resource is freely available at http://genome.igib.res.in/hgvtb/index.html. Using integrative analysis, we have shown that the disease associated variants are selectively enriched in the immune signalling pathways which are crucial in the pathophysiology of TB.

**Database URL:** http://genome.igib.res.in/hgvtb/index.html

## Introduction

Tuberculosis (TB) is an infectious disease caused by *Mycobacterium tuberculosis* (*Mtb*), an air-borne, nosocomial, gram positive and acid fast bacterium (1)*.* Nearly, one-third of the world’s population is estimated to be infected with this pathogen (2). The disease has emerged as one of the major causes of mortality and morbidity in the developing world (1, 3). It has been estimated that 8.8 million new cases of TB have been reported and ∼1.1 million affected individuals died in 2010. Majority of people infected with *M. tuberculosis* have latent infection with no evidence of clinical symptoms, but ∼10% of infected individuals develop clinical symptoms (1). Although the precise factors influencing the disease predisposition have not been well studied, several of them, such as pathogen virulence (4), host nutrition (5) and host genetic factors (6) have been implicated in causing the disease.

Differences in the disease susceptibility observed among different human populations followed by twin studies (7) suggested host genetic factors could at least in part influence predisposition to TB. In the early years, numerous studies aimed at understanding the genetic susceptibility to TB have been performed and extensively reviewed. However, they have largely been ambiguous (8). The first clear role of genetic factors in TB susceptibility was suggested through the pivotal experimental work of Lurie (9). Studies on different ethnic groups (10) and twins (7) provided additional evidence suggesting a larger role of host genetic factors in determining susceptibility to *M. tuberculosis* infection and progression to disease. More genetic associations have been shown in very recent years through approaches involving candidate genes (11–17). The use of genome-wide approaches also revealed significant genetic associations to TB infection in very recent years (18).

A number of distinct approaches and studies on diverse populations and ethnic groups have revealed genetic associations with respect to pathogenesis and outcome of TB. Though the data are available in the public domain, they are in disparate formats. A systematic attempt to collect, curate and perform integrative analysis of all the data on genetic factors, which influence susceptibility and outcome of TB could provide immense insights into the major pathways and mechanisms involved in the pathogenesis of TB and also open new avenues of investigation.

We have systematically collected evidence on host genetic associations with TB from peer reviewed literature and compiled them into a comprehensive and easily searchable online resource on human genes and genetic variants associated with TB (HGV&TB). The resource hosts information on 307 genetic variants from 162 studies. It provides a standardized view of genes and genetic variants, closely integrated with other online resources for gene and variant function analysis. For ease of future integration efforts, the genetic variant annotations in the resource have been standardized and conform to the recommendations of the Human Genome Variation Society (HGVS) (19) and recommendations for curation of Locus Specific Databases (20). Similarly, the gene names conform to the Human Gene Nomenclature Committee recommendations.

## Methods

### Data and resources

An exhaustive literature search was performed to retrieve all available evidences in the literature documenting association of the host genetic variability with TB. For the literature review, PubMed was searched using the keywords, such as ‘susceptibility’, ‘SNPs’, ‘variants’ and ‘genetics’ in combination with ‘TB’. Data for each of the associated variant were manually curated and classified. Gene annotations were mapped and standardized according to the Human Gene Nomenclature Committee. Genetic variants were also remapped and standardized to conform to the recommendations of the HGVS (19). The data were distributed among the annotators and collected on a shared document system implemented in Google Drive. Individual research papers depicting sequence variations and their association with TB were systematically referred back. The Mutalyzer 2.0 β-8 (21) (HGVS nomenclature version 2.0) was used to further check the entries made. The dbSNP was used for scrutiny of the IDs of the variant data. HGVS values were obtained from Mutalyzer 2.0 and dbSNP. Genomic variant change and location were uploaded directly from literature for the variations whose RSID’s were not available in dbSNP. All variants were cross checked and published only when no discrepancy was observed in the entries.

The sets of annotations were independently scrutinized manually by a team of database curators. Further in-depth bioinformatics analysis was performed using computational tools [SIFT (22) and PolyPhen2 (23)] to comprehend the potential biological significance of the variants and the genes that harbour them. The functional enrichment analysis was performed using the DAVID Bioinformatics Resource v6.7 (24). Disease classes and interaction pathways of the genes, which have been associated with genetic susceptibility to TB were analysed. All *P*-values were reported after the Bonferroni correction for multiple testing. Assessment of functional interaction of genes was performed using the STRING v.9.0 (Search Tool for the Retrieval of Interacting Gene/Proteins) (25).

### Database construction and features

The HGV&TB database was built in MySQL, and the browsable interface was created in HTML and Perl/CGI. Information for each mutation was compiled in annotation tables and made available through the searchable web interface. The database was built considering data interoperability and recommendations for curation of data using the guidelines provided by HUGO Gene Nomenclature Committee (HGNC) (26). For each mutation, information is provided at the molecular level, such as DNA change, exon, predicted amino acid change, type of mutation, reported and concluded pathogenicity, source of material, technique used and unique database ID. The gene and variant annotations comply with the HGNC recommendations. A brief citation of the source manuscript is also available in the database.

### Annotation of the variations

Two independent methods SIFT and PolyPhen2 were used to annotate the pathogenicity of the variants. While SIFT annotates the variants as tolerated and deleterious, PolyPhen2 uses the terms—benign, possibly damaging and probably damaging. The variants were annotated independently by each of the method, and a consensus was derived for the annotations. For each variant, a combination of annotations as tolerated and benign was considered and reported as ‘non-pathogenic’, tolerated and possibly damaging was reported as ‘probably pathogenic’ and deleterious or probably damaging was reported as ‘pathogenic’. The annotations of the function and gene interactions were analysed using two popular online tools, DAVID (24) and STRING (25), respectively. Allele frequency of the variants was retrieved from the HapMap (27) for each variation (28).

## Results and discussion

### Data summary

HGV&TB database harbours data for human genetic variations associated with susceptibility to different forms of TB, such as general TB, pulmonary, extra-pulmonary, pleural, miliary, spinal, cavitary, paediatric, meningeal and HIV-associated various forms of TB. The database hosts gene information on 98 genes and 307 variants. Of the total number of genes, 7 belong to the HLA class of genes, whereas 91 belong to non-HLA genes. The non-HLA genes including *CCL1, CCL2, CCL5, IFNG, IFNGR1,IFNGR2, IL10, IL12RB1, MBL2, NOS2, P2RX7, SLC11A1 (NRAMP1), SP110, TLR2, TLR4, TNF (TNFA)* and *VDR* have a large number of variants associated with TB. In addition, information on associated variants in HLA genes *HLA-A, B, C, DPB1, DQA1, DQB1* and *DRB1*, which have been extensively shown to be associated with TB in multiple studies, have also been indexed in the database.

**Figure 1.**
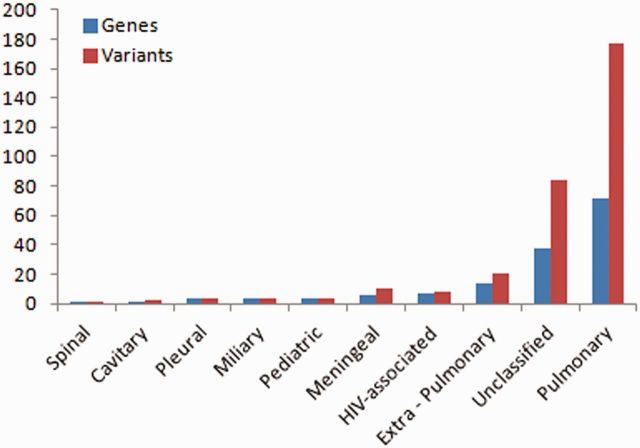
Number of variations having different phenotypic associations.

Of the total number of variants, 32 variants fall in this class of genes, from over 14 studies. A total of 72 genes and 177 variants were associated with pulmonary TB, whereas the rest were associated with other forms of TB including extra-pulmonary (14 genes, 20 variants), pleural (3 genes, 3 variants), miliary (4 genes, 4 variants), spinal (1 gene, 1 variant), cavitary (1 gene, 2 variants), paediatric (4 genes, 4 variants), meningeal (6 genes, 10 variants), HIV-associated (7 genes, 8 variants) and some were unclassified (38 genes, 84 variants) (Figure 1).

In HGV&TB, all genes and variants are indexed by unique database identifiers (HGVID). The resource could be searched using a variety of identifiers including HGVID, gene name, RSID, PMID, technique, template, geographic location, phenotype and concluded pathogenicity. Associated information including the HGVS nomenclature for the variants, the dbSNP RSID, genomic location and pathogenicity status of the variant as described in the primary literature have been included for each variant. In cases where the dbSNP RSIDs were not available, the variant change and position were obtained directly from the literature and compared with the databases using Mutalyzer. Supplementary Table S1 lists all the associated gene variant RSID’s of the respective genes, and Supplementary Table S2 lists the variant change and position of gene variants, along with associated literature reference where dbSNP RSID’s were not available.

Haplotypes, set of variations located on the same chromosome, are considered to be better determinants for establishing phenotypic association than single nucleotide variations (29). Thus, in addition to SNVs, we have also compiled information of haplotypes showing significant associations. The HGV&TB database contains information on 75 haplotypes in 37 genes (Supplementary Table S2). For example, SLC22A5 haplotype-c.652+77A>G-c.1052+237T>C-c.1053-550G>C was found to confer disease susceptibility in Thai trio family study only (30). Other genes, such as *CCL5, CTSZ, IL12RB1, IRGM, MBL2, SP110, TIRAP, TLR1* and *VDR* show both independent and haplotypic disease effect in various populations. In addition, gene studies in various Chinese populations revealed that variants could confer TB susceptibility, both when present individually and when present in the form of haplotype. Variants in *AKT1* gene affects pulmonary TB susceptibility in Chinese Han population both individually and in the form of a haplotype c.175+18C>T (rs3730358)-c.726G>A (rs1130233) (31). Similar pathogenesis pattern is observed in *BTNL2* gene (individual variant: rs3763313, rs9268494, rs9268492; haplotype: rs9268492-rs3763313-rs9268494-rs9405098-rs3763317-rs2076530) (32) and *MARCO* gene (individual variant: rs17009726, haplotype1: rs17009726-rs2278588 and haplotype2: rs17795618-rs1371562-rs6761637-rs2011839) (33).

### Analysis of genomic loci and gene position for associated variations

**Figure 2.**
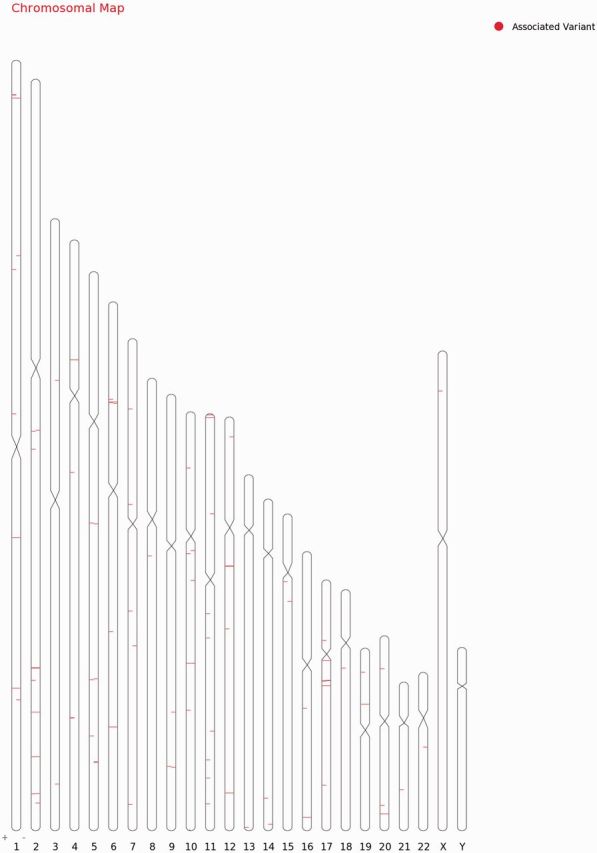
Chromosomal map showing the genomic loci and gene position mapping of the 255 variants with RSID’s.

**Figure 3.**
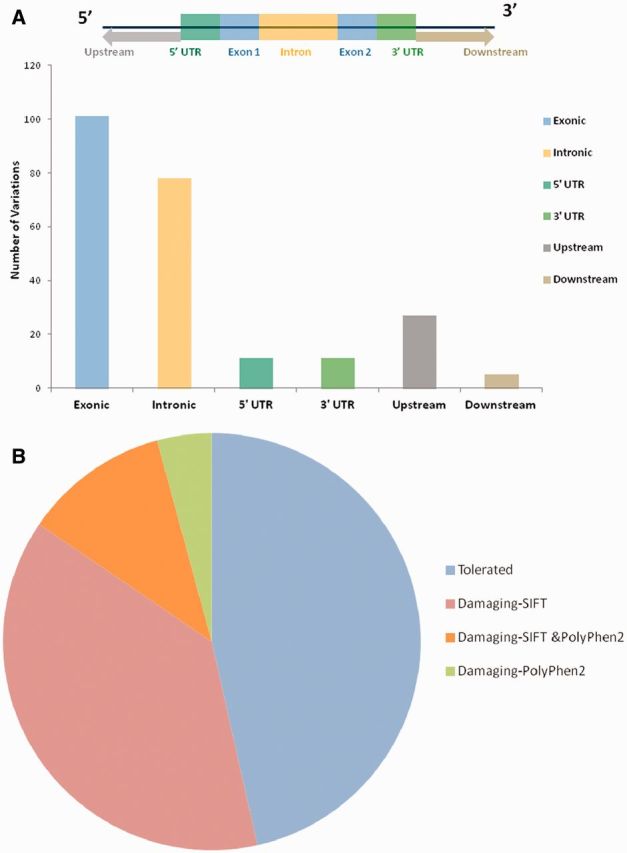
(A) Genomic locations of all the variations uploaded in HGV&TB. Total 307 variants are uploaded in HGV&TB, 101 mapped in exonic region while 78 mapped to the intronic and 11 to the 3′ UTR and 5′ UTR, respectively. Five mapped to the downstream regions of genes, whereas 27 mapped to the upstream regions of genes. (B) Potentially damaging exonic SNPs: combined results of SIFT and PolyPhen 2 concluded 48.39% (30/62) of the exonic SNPs to have a probably damaging functional role.

For each variant in all 98 genes, specific genomic location was determined from literature and public databases, such as dbSNP (34) and Ensembl (35). The chromosomal map of the variants is depicted in Figure 2. Of the total number of variations, 101 mapped to the coding sequence, 78 to the intronic region, and 38 in the intergenic region, 11 mapped to the 3′ untranslated regions and 11 to the 5′ untranslated regions. An additional 27 variants mapped to upstream and 5 variants to downstream regions of the genes. Apart from these, 10 variations are haplotypes and a total of 11 fall in the splicing sites or within the intronic, exonic and 5′-UTR of ncRNA. Fifteen variations of the total of 307 have not been assigned any genomic loci (Figure 3A; Supplementary Table S4). A total of 197 variants did not fall in close proximity to protein-coding genes (2 kb from TSS). Of these, 129 mapped to potential long-non-coding RNAs. The genomic loci and gene position mapping of the variants are summarized in Figure 2.

### Functional consequences of the variations as predicted by PolyPhen2 and SIFT

Apart from the reported pathogenicity of each of the variants, we performed an independent analysis of the potential functional consequences of each of the variations. We used two independent tools SIFT and PolyPhen2 for the analysis of the functional consequences of the variants in the database. Both methods have been extensively used in the past for annotation of deleterious effects of variations on protein structure and thereby the function (36). Of the total number of 101 variants which mapped to protein-coding genes, 27 (out of 60 predictions) and 11 (out of 46 predictions) were predicted to be deleterious by SIFT and PolyPhen2, respectively (Figure 3B). A total number of eight variants were in consensus predicted to be deleterious by both the tools (Supplementary Table S5; Supplementary Figure S1).

### Associations of variants with other diseases and/or traits

Many genes reported to be associated with TB are also found to be associated with other traits and conditions (Supplementary Table S1). For example, CHIT1 genomic variation is found to be associated with atopy, allergic rhinitis, contact dermatitis, food or drug allergy and asthma. Similarly, variants in other genes, such as BTNL2, CD209-promoter, CISH, CR-1, IL12A, MBL2, TLR8 were also found to be involved in various diseases. BTNL2 polymorphism has been recently associated with inflammatory autoimmune diseases, such as sarcoidosis and also associated with leprosy. Likewise, the CD209—promoter polymorphism—336A/G is associated with human susceptibility to dengue and HIV-1 besides TB. A complete list of disease classes to which each gene has been associated is presented in Supplementary Table S3.

### Functional interactions between associated genes

The genes harbouring associated variants were further analysed for mutual interactions using online tools available at DAVID Bioinformatics Resource v.6. (24). Functional annotation analysis from DAVID bioinformatics resource revealed that most of the genes formed close gene–gene interactions. This was observed from interactions based on direct or physical contact or interactions deduced using text-mining, gene context and high-throughput experiments including correlations in expression. This precisely points to the close biological context in the functional organization of the genes. A close analysis of the genes also revealed their enrichment in cell signalling pathways, especially in the Toll-like receptor signalling, cytokine–cytokine receptor interaction and the *JAK-STAT* pathways. No functional interaction was observed between the genes reported in the catalogue of published genome wide association studies for TB. The pathways enriched have been summarized in Table 1. A highly connected network of all the genes associated with TB was generated by STRING v.9.0 with almost every gene linked to more than one other gene (Supplementary Figure S2).

**Table 1.** Pathway enrichment of TB susceptibility genes obtained from DAVID Bioinformatics Resource v6.7l

Pathway	Count	Genes	*P*-value	Fisher extract
Cytokine–cytokine receptor interaction	26	*CCL1, IL1R1, TNF, CCL2, IL18, CCL5, CXCL12, IL10, IL12RB2, TNFRSF1A, TNFRSF1B, IL12RB1, IL10RA, IFNG, IL1B, IFNGR2, IFNGR1, LTA, IL1A, IL4, IL6, IL23R, IL8, IL6R, IL12B, IL2*	4.54E-16	5.60E-17
Type I diabetes mellitus	13	*HLA-DQB1, TNF, HLA-DRB1, HLA-A, HLA-C, HLA-B, HLA-DQA1, IFNG, IL1B, HLA-DPB1, IL12B, LTA, IL1A, IL2*	7.96E-14	2.50E-15
Allograft rejection	12	*IL4, HLA-DQB1, TNF, HLA-DRB1, HLA-A, HLA-C, HLA-B, IL10, HLA-DQA1, IFNG, IL12B, HLA-DPB1, IL2*	4.11E-13	1.20E-14
Graft-versus-host disease	12	*HLA-DQB1, IL6, TNF, HLA-DRB1, HLA-A, HLA-C, HLA-B, HLA-DQA1, IFNG, IL1B, HLA-DPB1, IL1A, IL2*	1.11E-12	3.50E-14
Toll-like receptor signalling pathway	16	*IL6, TNF, IL8, TOLLIP, TLR1, TIRAP, TLR2, TLR4, TLR6, CCL5, TLR8, TLR9, AKT1, IL1B, IL12B, CD14*	1.51E-12	1.10E-13
Jak-STAT signalling pathway	15	*IL4, IL6, IL23R, IL6R, CISH, IL10, AKT1, IL12RB2, IL12RB1, IL10RA, IFNG, IL12B, IFNGR2, IFNGR1, IL2*	8.06E-09	1.00E-09
Intestinal immune network for IgA production	9	*HLA-DQB1, IL4, IL6, HLA-DRB1, HLA-DPB1, CXCL12, HLA-DQA1, IL10, IL2*	1.74E-07	1.10E-08
Autoimmune thyroid disease	9	*HLA-DQB1, IL4, HLA-DRB1, HLA-A, HLA-C, HLA-B, HLA-DPB1, HLA-DQA1, IL10, IL2*	2.41E-07	1.50E-08
Hematopoietic cell lineage	10	*IL4, CR1, IL1R1, IL6, TNF, HLA-DRB1, IL1B, IL6R, CD14, IL1A*	1.42E-06	1.50E-07
Asthma	7	*HLA-DQB1, IL4, TNF, HLA-DRB1, HLA-DPB1, HLA-DQA1, IL10*	1.56E-06	6.90E-08
Antigen processing and presentation	9	*HLA-DQB1, HLA-DRB1, TAP2, TAP1, HLA-A, HLA-C, HLA-B, HLA-DPB1, HLA-DQA1, LTA*	1.07E-05	1.20E-06
NOD-like receptor signalling pathway	8	*NOD2, IL6, TNF, CCL2, IL8, IL18, IL1B, CCL5*	1.39E-05	1.30E-06
Systemic lupus erythematosus	7	*HLA-DQB1, TNF, HLA-DRB1, IFNG, HLA-DPB1, HLA-DQA1, IL10*	0.001789	3.10E-04
Viral myocarditis	6	*HLA-DQB1, HLA-DRB1, HLA-A, HLA-C, HLA-B, HLA-DPB1, HLA-DQA1*	0.002272	3.30E-04
Apoptosis	6	*AKT1, TNFRSF1A, IL1R1, TNF, IL1B, IL1A*	0.005485	9.90E-04
Linoleic acid metabolism	4	*CYP3A4, CYP3A5, CYP2C19, CYP2E1*	0.00566	4.70E-04
Prion diseases	4	*IL6, IL1B, CCL5, IL1A*	0.010592	1.10E-03
Adipocytokine signalling pathway	5	*AKT1, TNFRSF1A, TNFRSF1B, TNF, MTOR*	0.011329	1.90E-03
T-cell receptor signalling pathway	6	*IL4, AKT1, TNF, IFNG, IL10, IL2*	0.013395	3.00E-03
Cell adhesion molecules (CAMs)	6	*HLA-DQB1, HLA-DRB1, HLA-A, HLA-C, HLA-B, HLA-DPB1, HLA-DQA1*	0.029227	8.00E-03
Natural killer cell-mediated cytotoxicity	6	*TNF, IFNG, HLA-A, HLA-C, HLA-B, IFNGR2, IFNGR1*	0.030067	8.30E-03
Cytosolic DNA-sensing pathway	4	*IL6, IL18, IL1B, CCL5*	0.035259	6.00E-03
Metabolism of xenobiotics by cytochrome P450	4	*CYP3A4, CYP3A5, CYP2C19, CYP2E1*	0.043919	8.10E-03
Drug metabolism	4	*CYP3A4, CYP3A5, CYP2C19, CYP2E1*	0.047655	9.10E-03
RIG-I-like receptor signalling pathway	4	*TNF, ATG5, IL8, IL12B*	0.066314	1.50E-02
Regulation of autophagy	3	*ATG4C, ATG5, IFNG*	0.077058	1.10E-02
Chemokine signalling pathway	6	*CCL1, AKT1, CCL2, IL8, CCL5, CXCL12*	0.098909	3.80E-02

The table summarizes the number of genes involved in various biological pathways, according to KEGG bioinformatics resource. Fifty-four genes, of the total 98 genes in HGV&TB, are categorized under 27 different pathways. Count indicates the exact number of genes involved in a particular pathway, and various statistical values are provided in the table. *P*-value here refers to the modified Fisher Exact *P*-value (EASE score); smaller the score, more enriched classification.

We also found evidence of gene interaction from the haplotypes associated with susceptibility to TB. Few variations in genes showed association, in concurrence with the presence of other variants of a different set of genes. For example, *TLR6* gene variant (rs5743810) was not independently associated with TB, while in combination with *TLR1* variants (rs4833095 and rs76798247); it was found to affect susceptibility to TB in African American population (37). Some disease susceptible gene variations are also found in diverse human populations, both independently and in association with variants of same or other genes. *TNF alpha* (TNF gene) variants (-238G/A, -308G->A, -836 A/C) were associated with various forms of TB in diverse populations either independently (38–44) or in combination with variants in *TLR4* gene [rs7791836 (*TNF*)-rs1399431 (*TLR4*)] (45). Similarly, variations in other genes, such as *CCL2* (46–50) and *PSMB48* (51) were jointly associated with susceptibility to TB. It was also observed that variations in *NOS2A, TLR4* and *IFNGR1* were susceptible in different populations, both individually and in association with variants of same as well as different genes. But in African populations they showed strong gene–gene interactions leading towards various forms of TB.

### Analysis of population frequencies of variations in HGV&TB

Independent evaluation of the variations in the HGV&TB database revealed that maximum number of variants have been discovered in African population (*N* = 90) followed by Chinese (*N* = 74) and Indian (*N* = 69) (Supplementary Table S6). Most of the reports of genes and genetic variants were confined to one population or ethnic group, barring a handful of genes, such as *IFNG*, *IFNGR1*, *SLC11A1* and *VDR* which have been shown to be associated in a number of populations, suggesting a robust association.

In addition, allele frequencies of variations in the database were independently analysed in the world populations, using data from the HapMap project (Supplementary Figure S3; Supplementary Table S7). Additional analysis was performed on the basis of integrated haplotype score (iHS), a statistical parameter to detect evidence for positive selection. iHS data corresponding to different populations and chromosome for Hapmap phase 2 was downloaded and parsed for the entire variant dataset with respect to rsIDs. A total of 20 variants showed evidence of selection (iHS < -2) in populations (CEU, YRI) (Supplementary Table S8).

### Database usage and navigation

The interface of the database has been built as a user friendly GUI wherein the homepage provides a brief summary and a search box for different query options. Each query directs the users to a table reporting all the genes and associated variations with their corresponding *P*-value, odds ratio, geographic location, pathogenicity and reference of the study. HGVID (HGV&TB identifiers) and rsID on this page are linked to a detailed report of the respective query in context to gene, variant, study details and external links. The ‘Gene’ panel reports the name of the gene, haplotype reported (if any) and genomic location of the gene and the variant. The ‘Variant’ panel provides a description about the type of the variant, reported phenotype, *P*-value and odds ratio, the reported and concluded pathogenicity and HGVS values corresponding to the variant. The ‘Details’ panel reports the details of the study which quotes the respective TB susceptible variation in context to the detection template, detection technique and the origin, ethnicity and geographic locations of the population under study. External links to dbSNP, PUBMED, UCSC (52) and Gene Card (53) have also been provided.

### Discussion and future perspective

The advent of newer technologies for analysing genetic variations, including whole-genome sequencing methods, would enable researchers to query genomic signatures and to fine-map functional variations in genes previously shown to be associated with disease susceptibility. This would also unearth more genetic loci which confer susceptibility to various forms of TB. Systematic curation of such variations and their association from literature and sources of evidence needs to be done on a continuous basis. The involvement of the community would help achieve this goal much faster and more accurately. HGV&TB provides a starting point towards involving a larger community of researchers in the field who would on one end contribute their time and expertise curating variants, and on the other end see how these genetic variations could potentially be used in diverse clinical applications. In addition to the data being up-to-date, it is also important to ensure that the data are inter-operable with platforms and systems for analysis. The raw data have been provided on the server which can be used to perform meta-analysis to identify patterns and interesting relationships in context of multiple studies. To this end we foresee co-operation and collaboration with systems which facilitate exchange of data between resources and tools like Cafe Variome, where data are shared within different laboratories having common interest or with the wider world (http://www.cafevariome.org/). We also foresee the potential integration of the data in resources which could enable automated analysis of whole-genome sequencing data, including data from personal genomes.

## Funding

Financial support to Y.H. from Council of Scientific & Industrial Research (Grant Code: HCP0001) is acknowledged. DB acknowledges a senior research fellowship from Council of Scientific and Industrial Research, India.

## Supplementary Material

Supplementary Data
