# Supplementary material for: HGV&TB: a comprehensive online resource on human genes and genetic variants associated with tuberculosis
Source: Database (Oxford). 2014 Dec 13;2014:bau112. doi: 10.1093/database/bau112 (PMC5630898; doi:10.1093/database/bau112)
Supplement: Supplementary Data [file bau112_Supplementary_Data.zip › Supplementary_Table_3.docx]

**Supplementary Table 3:**

**Classes of Diseases mapping to Genes involved in Genetic Susceptibility to tuberculosis:** The Analysis was performed using DAVID Bioinformatics Resource v6.7. The table summarizes the number of genes associated with various disease classes. **82** genes, of the total **98** genes in HGV&TB, are categorized under **17** different disease classes, referenced from Genetic Association Database. Count indicates the exact number of genes involved in a particular disease class and ‘%’ column is the calculation of percentage of the involved genes and total genes. In support, various statistical values are provided in the table. P-value here refers to the modified Fisher Exact P-value (EASE score); smaller the score, more enriched classification.

| **Disease** | **Count** | **%** | **Genes** | **P-Value** | **Fold Enrichment** | **Bonferroni** |
| --- | --- | --- | --- | --- | --- | --- |
| INFECTION | 61 | 63.54167 | CYP3A4, CYP3A5, CYP2C19, IL18, TLR1, TLR2, TIRAP, PTPN22, TLR4, TLR6, CXCL12, IL10, TLR8, TLR9, NOD2, IFNG, IL1B, NOS2, IFNGR2, IFNGR1, LTA, IL1A, BTNL2, HLA-A, HLA-C, HLA-B, CYP2E1, HLA-DQA1, IL12B, HLA-DQB1, CCL1, MBL2, IL1R1, CCL2, TNF, HLA-DRB1, SP110, SFTPA1, CCL5, CHIT1, MIF, IL12RB2, TNFRSF1A, SLC11A1, VDR, TNFRSF1B, IL12RB1, TAP2, IL10RA, TAP1, HLA-DPB1, IL4, CR1, IL6, IL8, IL1RN, PSMB8, P2RX7, CD209, SFTPA2, CD14, IL2 | 9.69E-49 | 8.130311243 | 1.74E-47 |
| IMMUNE | 67 | 69.79167 | CYP3A4, CYP3A5, CYP2C19, TOLLIP, IL18, TLR1, TLR2, PTPN22, TLR4, JAG1, TLR6, CXCL12, IL10, TLR9, NOD2, IFNG, IL1B, NOS2, SLC22A5, IFNGR2, LTA, IL1A, IFNGR1, BTNL2, HLA-A, HLA-C, IL6R, HLA-B, HLA-DQA1, IL12B, SLC40A1, HLA-DQB1, CCL1, MBL2, IL1R1, CCL2, TNF, HLA-DRB1, SFTPA1, CCL5, CHIT1, MIF, IL12RB2, TNFRSF1A, SLC11A1, VDR, TNFRSF1B, IL12RB1, TAP2, IL10RA, TAP1, HLA-DPB1, IL4, IL6, CR1, IL23R, IL8, IL1RN, PSMB8, P2RX7, CD209, IRF8, EBF1, SFTPA2, ALOX5, ATG16L1, CD14, IL2 | 5.22E-30 | 3.364122917 | 9.39E-29 |
| REPRODUCTION | 33 | 34.375 | HLA-DQB1, CYP3A4, CYP3A5, TNF, HLA-DRB1, CYP2C19, IL18, TLR2, TLR4, CCL5, IL10, TNFRSF1A, VDR, TNFRSF1B, NOD2, TAP2, TAP1, IFNG, IL1B, NOS2, HLA-DPB1, IL1A, LTA, IL4, IL6, IL1RN, HLA-A, HLA-C, CYP2E1, HLA-B, IL6R, HLA-DQA1, CD14, IL2 | 1.23E-16 | 5.338338285 | 2.00E-15 |
| UNKNOWN | 32 | 33.33333 | HLA-DQB1, CYP3A4, MBL2, IL1R1, TNF, CCL2, HLA-DRB1, CYP2C19, SFTPA1, CCL5, IL10, TNFRSF1A, TNFRSF1B, TAP1, IFNG, IL1B, NOS2, HLA-DPB1, IL1A, LTA, IL4, IL6, IL8, IL1RN, HLA-A, HLA-C, CYP2E1, HLA-B, IL6R, HLA-DQA1, SFTPA2, CD14, IL2 | 1.82E-16 | 5.509826067 | 4.00E-15 |
| NORMALVARIATION | 35 | 36.45833 | HLA-DQB1, CYP3A4, MBL2, CYP3A5, IL1R1, TNF, HLA-DRB1, CYP2C19, TLR2, TLR4, SFTPA1, IL10, MIF, TLR9, IL12RB2, TNFRSF1A, VDR, TNFRSF1B, IFNG, IL1B, NOS2, HLA-DPB1, MC3R, IL1A, LTA, IL4, IL6, IL8, IL1RN, HLA-A, HLA-C, CYP2E1, HLA-B, HLA-DQA1, CD209, IL2 | 4.01E-16 | 4.727760057 | 7.99E-15 |
| CANCER | 50 | 52.08333 | CYP3A4, CYP3A5, CYP2C19, IL18, TLR1, TLR2, TIRAP, TLR4, TLR6, CXCL12, IL10, TLR9, NOD2, IFNG, IL1B, NOS2, IFNGR2, LTA, IL1A, HLA-A, HLA-C, HLA-B, CYP2E1, HLA-DQA1, IL12B, SLC40A1, HLA-DQB1, IL1R1, TNF, CCL2, HLA-DRB1, SFTPA1, CCL5, TNFRSF1A, SLC11A1, VDR, TNFRSF1B, IL12RB1, TAP2, IL10RA, TAP1, HLA-DPB1, MRC1, IL4, IL6, IL8, IL1RN, P2RX7, ALOX5, CD14, IL2 | 1.84E-13 | 2.591629267 | 3.30E-12 |
| CARDIOVASCULAR | 47 | 48.95833 | HLA-DQB1, CYP3A4, CYP3A5, MBL2, IL1R1, CCL2, TNF, CYP2C19, HLA-DRB1, IL18, TLR2, PTPN22, TLR4, SFTPA1, CCL5, CXCL12, IL10, MIF, TLR9, TNFRSF1A, VDR, TNFRSF1B, IL10RA, IFNG, TAP1, IL1B, NOS2, SLC22A5, HLA-DPB1, IFNGR2, LTA, IFNGR1, IL1A, IL4, CR1, IL6, IL1RN, HLA-A, HLA-C, CYP2E1, HLA-B, HLA-DQA1, PSMB8, SFTPA2, IL12B, ALOX5, CD14, IL2 | 2.02E-13 | 2.768819074 | 3.63E-12 |
| OTHER | 49 | 51.04167 | CYP3A5, CYP2C19, IL18, TLR2, PTPN22, TLR4, CXCL12, IL10, TLR9, NOD2, IFNG, IL1B, SLC22A5, NOS2, IFNGR1, LTA, IL1A, HLA-A, HLA-C, CYP2E1, HLA-B, HLA-DQA1, IL12B, SLC40A1, HLA-DQB1, MBL2, TNF, CCL2, HLA-DRB1, SFTPA1, CCL5, MIF, TNFRSF1A, VDR, TNFRSF1B, TAP2, IL10RA, TAP1, HLA-DPB1, MC3R, IL4, CR1, IL6, IL8, IL1RN, PSMB8, P2RX7, SFTPA2, CD14, IL2 | 5.24E-13 | 2.583183484 | 9.44E-12 |
| RENAL | 25 | 26.04167 | CYP3A4, HLA-DQB1, CYP3A5, IL1R1, TNF, CCL2, HLA-DRB1, TLR4, CCL5, IL10, MIF, VDR, IFNG, IL1B, HLA-DPB1, NOS2, LTA, IL4, IL6, CR1, IL8, IL1RN, HLA-DQA1, CD14, IL2 | 1.05E-12 | 5.634609698 | 1.88E-11 |
| PHARMACOGENOMIC | 32 | 33.33333 | HLA-DQB1, CYP3A4, CYP3A5, TNF, CCL2, HLA-DRB1, CYP2C19, IL18, PTPN22, TLR4, IL10, MIF, VDR, NOD2, TAP2, IFNG, IL1B, IFNGR2, IFNGR1, LTA, IL4, IL6, IL8, IL1RN, HLA-A, HLA-C, CYP2E1, HLA-B, PSMB8, HLA-DQA1, IRF8, IL12B, ALOX5 | 4.15E-12 | 3.890271132 | 7.46E-11 |
| NEUROLOGICAL | 40 | 41.66667 | HLA-DQB1, CYP3A4, CYP3A5, IL1R1, CCL2, TNF, CYP2C19, HLA-DRB1, IL18, TLR4, JAG1, CCL5, CXCL12, IL10, MIF, TNFRSF1A, VDR, SLC11A1, TNFRSF1B, IL12RB1, TAP2, IFNG, IL1B, NOS2, IFNGR2, IFNGR1, IL1A, LTA, IL4, IL6, IL8, IL1RN, HLA-A, HLA-C, CYP2E1, HLA-B, HLA-DQA1, PSMB8, ALOX5, CD14, IL2 | 7.91E-11 | 2.771566221 | 1.42E-09 |
| HEMATOLOGICAL | 20 | 20.83333 | HLA-DQB1, IL4, CYP3A4, CR1, TNF, HLA-DRB1, IL1RN, HLA-A, TLR4, HLA-C, CYP2E1, HLA-B, CXCL12, IL10, TLR9, VDR, TNFRSF1A, TNFRSF1B, IFNG, IL1B, NOS2 | 5.95E-10 | 5.572002924 | 1.07E-08 |
| AGING | 14 | 14.58333 | IL4, IL6, TNF, IL8, IL18, IL1RN, HLA-A, TLR4, IL10, VDR, IFNG, IL1B, IL1A, IL2 | 1.50E-06 | 5.152824722 | 2.70E-05 |
| VISION | 18 | 18.75 | HLA-DQB1, MBL2, IL6, TNF, CCL2, HLA-DRB1, IL1RN, TLR4, CYP2E1, CCL5, TAP2, TAP1, IFNG, IL1B, HLA-DPB1, NOS2, IFNGR1, IL1A | 1.52E-06 | 3.861666198 | 2.74E-05 |
| METABOLIC | 40 | 41.66667 | HLA-DQB1, CYP3A4, MBL2, CYP3A5, IL1R1, TNF, CCL2, HLA-DRB1, CYP2C19, TLR2, PTPN22, TLR4, CCL5, IL10, MIF, AKT1, TNFRSF1A, VDR, TNFRSF1B, TAP2, IFNG, TAP1, IL1B, NOS2, MC3R, HLA-DPB1, IL1A, LTA, IL4, IL6, IL8, IL1RN, HLA-A, HLA-C, CYP2E1, HLA-B, IL6R, HLA-DQA1, LAMP1, SLC40A1, CD14 | 4.96E-06 | 1.910401003 | 8.92E-05 |
| CHEMDEPENDENCY | 11 | 11.45833 | AKT1, TNFRSF1A, TNF, IL8, PENK, IL1RN, NOS2, CYP2E1, JAG1, IL10, CD14 | 0.008862 | 2.550882263 | 0.148052366 |
| PSYCH | 22 | 22.91667 | HLA-DQB1, IL4, MBL2, IL6, IL1R1, TNF, CCL2, HLA-DRB1, CYP2C19, HLA-A, CYP2E1, IL10, HLA-DQA1, AKT1, P2RX7, PENK, TAP2, IL10RA, IL1B, MC3R, NOS2, IL2 | 0.023898 | 1.584569593 | 0.352988179 |
