# Supplementary material for: HGV&TB: a comprehensive online resource on human genes and genetic variants associated with tuberculosis
Source: Database (Oxford). 2014 Dec 13;2014:bau112. doi: 10.1093/database/bau112 (PMC5630898; doi:10.1093/database/bau112)
Supplement: Supplementary Data [file bau112_Supplementary_Data.zip › Supplementary_Table_5.docx]

**Supplementary Table 5: Functional consequences of the variations as predicted by PolyPhen2 and SIFT**

**SIFT and PolyPhen 2 combined results**. Only those SNPs are included which are common in both SIFT and PolyPhen 2 results.

**PloyPhen 2 and SIFT common SNPs:**

|  | **FUNCTIONAL ANNOTATION** | |
| --- | --- | --- |
| **rsid** | **SIFT** | **POLYPHEN** |
| rs3135932 | TOLERATED | possibly damaging |
| rs8177374 | TOLERATED | possibly damaging |
| rs11209026 | DAMAGING | probably damaging |
| rs1800450 | DAMAGING | probably damaging |
| rs1800451 | DAMAGING | probably damaging |
| rs2066844 | DAMAGING | probably damaging |
| rs3751143 | DAMAGING | probably damaging |
| rs5030737 | DAMAGING | probably damaging |
| rs5743708 | DAMAGING | probably damaging |
| rs8177400 | DAMAGING | probably damaging |
| rs11556887 | TOLERATED | probably damaging |

**PolyPhen 2 All Results**

| **rsid** | **acc** | **prediction** | **based_on** | **pph2_class** | **pph2_prob** | **pph2_FPR** | **pph2_TPR** | **pph2_FDR** |
| --- | --- | --- | --- | --- | --- | --- | --- | --- |
| rs1041981 | P01374 | benign | alignment_mz | neutral | 0.005 | 0.26 | 0.968 | 0.267 |
| rs1065761 | Q13231 | benign | alignment_mz | neutral | 0 | 1 | 1 | 0.575 |
| rs11209026 | Q5VWK5 | probably damaging | alignment | deleterious | 1 | 0.00026 | 0.00018 | 0.0109 |
| rs11235604 | Q8NAA4 | benign | alignment | neutral | 0.078 | 0.154 | 0.933 | 0.183 |
| rs1135216 | Q03518 | benign | alignment | neutral | 0 | 1 | 1 | 0.575 |
| rs1135791 | Q9HB58 | benign | alignment | neutral | 0 | 1 | 1 | 0.575 |
| rs1136450 | Q8IWL2 | benign | alignment | neutral | 0 | 1 | 1 | 0.575 |
| rs11568350 | Q9NP59 | benign | alignment | neutral | 0.01 | 0.226 | 0.96 | 0.242 |
| rs11575934 | P42701 | benign | alignment | neutral | 0 | 1 | 1 | 0.575 |
| rs17235409 | P49279 | benign | alignment | neutral | 0.078 | 0.154 | 0.933 | 0.183 |
| rs17886395 | Q8IWL1 | benign | alignment | neutral | 0 | 1 | 1 | 0.575 |
| rs1800450 | P11226 | probably damaging | alignment | deleterious | 1 | 0.00026 | 0.00018 | 0.0109 |
| rs1800451 | P11226 | probably damaging | alignment | deleterious | 0.994 | 0.0289 | 0.689 | 0.0537 |
| rs1864183 | Q9H0Y0 | benign | alignment_mz | neutral | 0.113 | 0.142 | 0.927 | 0.172 |
| rs1965708 | Q8IWL1 | benign | alignment | neutral | 0 | 1 | 1 | 0.575 |
| rs2066842 | Q9HC29 | benign | alignment | neutral | 0.017 | 0.205 | 0.954 | 0.225 |
| rs2066844 | Q9HC29 | probably damaging | alignment | deleterious | 0.998 | 0.0112 | 0.273 | 0.0274 |
| rs2228065 | P09917 | benign | alignment | neutral | 0 | 1 | 1 | 0.575 |
| rs2241880 | Q676U5 | benign | alignment | neutral | 0 | 1 | 1 | 0.575 |
| rs231775 | P16410 | benign | alignment | neutral | 0.007 | 0.246 | 0.965 | 0.257 |
| rs2476601 | Q9Y2R2 | benign | alignment | neutral | 0.029 | 0.183 | 0.948 | 0.207 |
| rs28371759 | P08684 | benign | alignment | neutral | 0.089 | 0.149 | 0.932 | 0.178 |
| rs3135932 | Q13651 | possibly damaging | alignment | deleterious | 0.754 | 0.0773 | 0.852 | 0.109 |
| rs34069356 | Q9UBR2 | benign | alignment | neutral | 0.001 | 0.852 | 0.994 | 0.514 |
| rs3734114 | Q9H0Y0 | benign | alignment_mz | neutral | 0.005 | 0.26 | 0.968 | 0.267 |
| rs3751143 | Q99572 | probably damaging | alignment | deleterious | 0.991 | 0.0326 | 0.711 | 0.0584 |
| rs375947 | P42701 | benign | alignment | neutral | 0 | 1 | 1 | 0.575 |
| rs3827103 | P41968 | benign | sequence annotation | neutral | 0.429 | 0.102 | 0.892 | 0.135 |
| rs3948464 | Q9HB58 | benign | alignment | neutral | 0 | 1 | 1 | 0.575 |
| rs401502 | P42701 | benign | alignment | neutral | 0.006 | 0.253 | 0.967 | 0.262 |
| rs482912 | Q9UQV4 | benign | alignment | neutral | 0 | 1 | 1 | 0.575 |
| rs4986790 | O00206 | benign | alignment | neutral | 0.026 | 0.188 | 0.949 | 0.211 |
| rs5030737 | P11226 | probably damaging | alignment | deleterious | 1 | 0.00026 | 0.00018 | 0.0109 |
| rs5743278 | Q9HC29 | benign | alignment | neutral | 0.04 | 0.173 | 0.944 | 0.199 |
| rs5743708 | O60603 | probably damaging | alignment | deleterious | 1 | 0.00026 | 0.00018 | 0.0109 |
| rs8177374 | P58753 | possibly damaging | alignment | neutral | 0.456 | 0.0995 | 0.888 | 0.132 |
| rs8177400 | P58753 | probably damaging | alignment | deleterious | 1 | 0.00026 | 0.00018 | 0.0109 |
| rs883541 | Q5MNZ9 | benign | alignment | neutral | 0 | 1 | 1 | 0.575 |
| rs9061 | Q9HB58 | benign | alignment | neutral | 0.012 | 0.219 | 0.958 | 0.236 |
| rs9323945 | Q96BY7 | benign | alignment | neutral | 0 | 1 | 1 | 0.575 |
| rs2229094 | P01374 | benign | alignment_mz | neutral | 0 | 1 | 1 | 0.575 |
| rs5743810 | Q9Y2C9 | benign | alignment | neutral | 0 | 1 | 1 | 0.575 |
| rs6761637 | Q9UEW3 | benign | alignment_mz | neutral | 0 | 1 | 1 | 0.575 |
| rs11556887 | Q9HB58 | probably damaging | alignment | deleterious | 0.998 | 0.0112 | 0.273 | 0.0274 |
| rs3802813 | P58753 | benign | alignment_mz | neutral | 0.003 | 0.556 | 0.981 | 0.39 |
| rs2076530 | Q9UIR0 | benign | alignment | neutral | 0 | 1 | 1 | 0.575 |

**SIFT All RESULTS**

| **SNP** | **Amino acid change** | **Protein ID** | **Amino Acid** | **Using orthologues in the protein alignment** | | | **Using homologues in the protein alignment** | | |
| --- | --- | --- | --- | --- | --- | --- | --- | --- | --- |
|  |  |  |  | [Prediction](http://sift.jcvi.org/www/dbsnp_example.html#Prediction) | **Score** | [Median info](http://sift.jcvi.org/www/dbsnp_example.html#Median_info) | [Prediction](http://sift.jcvi.org/www/dbsnp_example.html#Prediction) | **Score** | [Median info](http://sift.jcvi.org/www/dbsnp_example.html#Median_info) |
| rs1041981 | T60N | NP_000586 | T | TOLERATED | 0.91 | 2.42 | TOLERATED | 1 | 4.32 |
|  |  |  | N | TOLERATED | 0.51 |  | TOLERATED | 0.1 |  |
| rs1065761 | A442G | NP_003456 | A | TOLERATED | 0.45 | 2.26 | TOLERATED | 0.11 | 3.32 |
|  |  |  | G | TOLERATED | 1 |  | TOLERATED | 0.63 |  |
| rs1065761 | A442V | NP_003456 | A | TOLERATED | 0.45 | 2.26 | TOLERATED | 0.11 | 3.32 |
|  |  |  | V | TOLERATED | 0.11 |  | DAMAGING | 0.02 | *Warning! Low confidence.** |
| rs11209026 | R381Q | NP_653302 | R | TOLERATED | 1 | 3.16 | TOLERATED | 1 | 3.02 |
|  |  |  | Q | DAMAGING | 0.02 |  | TOLERATED | 0.08 |  |
| rs11235604 | R220W | NP_203746 | R | TOLERATED | 0.82 | 1.88 | TOLERATED | 1 | 3.13 |
|  |  |  | W | DAMAGING | 0 |  | DAMAGING | 0 |  |
| rs1135216 | D697G | NP_000584 | D | DAMAGING | 0.03 | 2.47 | DAMAGING | 0.05 | 3.71 |
|  |  |  | G | TOLERATED | 1 |  | TOLERATED | 1 | *Warning! Low confidence.** |
| rs1135791 | M529T | NP_001171944 | M | TOLERATED | 0.25 | 2.69 | TOLERATED | 0.26 | 2.37 |
|  |  |  | T | TOLERATED | 0.46 |  | TOLERATED | 0.38 |  |
| rs1136450 | L65V | NP_001087239 | L | TOLERATED | 0.78 | 1.64 | TOLERATED | 0.3 | 4.12 |
|  |  |  | V | TOLERATED | 0.63 |  | TOLERATED | 1 |  |
| rs11568350 | Q248H | NP_055400 | Q | TOLERATED | 1 | 1.9 | TOLERATED | 1 | 2.76 |
|  |  |  | H | TOLERATED | 0.12 |  | TOLERATED | 0.24 |  |
| rs11575934 | Q214R | NP_005526 | Q | TOLERATED | 0.3 | 2.39 | TOLERATED | 0.3 | 2.12 |
|  |  |  | R | TOLERATED | 0.36 |  | TOLERATED | 0.36 |  |
| rs17235409 | D543N | NP_000569 | D | TOLERATED | 0.86 | 3.38 | TOLERATED | 1 | 4.1 |
|  |  |  | N | TOLERATED | 0.2 |  | DAMAGING | 0.03 | *Warning! Low confidence.** |
| rs17886395 | A91P | NP_001092138 | A | TOLERATED | 0.3 | 1.75 | TOLERATED | 0.07 | 3.91 |
|  |  |  | P | TOLERATED | 1 |  | TOLERATED | 1 |  |
| rs1800450 | G54D | NP_000233 | G | TOLERATED | 1 | 1.87 | TOLERATED | 1 | 3.5 |
|  |  |  | D | DAMAGING | 0 |  | DAMAGING | 0 | *Warning! Low confidence.** |
| rs1800451 | G57E | NP_000233 | G | TOLERATED | 1 | 1.87 | TOLERATED | 1 | 3.5 |
|  |  |  | E | DAMAGING | 0 |  | DAMAGING | 0 | *Warning! Low confidence.** |
| rs1864183 | T212M | NP_001124500 | T | TOLERATED | 0.87 | 1.8 | TOLERATED | 1 | 2.58 |
|  |  |  | M | TOLERATED | 0.13 |  | TOLERATED | 0.25 |  |
| rs1965708 | Q223K | NP_001092138 | Q | TOLERATED | 0.17 | 1.97 | TOLERATED | 1 | 4.19 |
|  |  |  | K | TOLERATED | 0.34 |  | TOLERATED | 0.36 |  |
| rs2066842 | P268S | NP_071445 | P | TOLERATED | 1 | 2.6 | TOLERATED | 1 | 3.7 |
|  |  |  | S | TOLERATED | 0.33 |  | TOLERATED | 0.06 |  |
| rs2066844 | R702W | NP_071445 | R | TOLERATED | 1 | 2.64 | TOLERATED | 0.8 | 3.52 |
|  |  |  | W | DAMAGING | 0 |  | DAMAGING | 0 | *Warning! Low confidence.** |
| rs2071543 | Q49K | NP_683720 | Q | TOLERATED | 0.54 | 3.15 | TOLERATED | 1 | 4.32 |
|  |  |  | K | TOLERATED | 0.12 |  | DAMAGING | 0.03 | *Warning! Low confidence.** |
| rs2228065 | E254K | NP_000689 | E | TOLERATED | 1 | 1.8 | TOLERATED | 0.89 | 2.95 |
|  |  |  | K | TOLERATED | 0.74 |  | TOLERATED | 1 |  |
| rs2228570 | M1K | NP_000367 | M | TOLERATED | 1 | 4.32 | TOLERATED | 1 | 4.32 |
|  |  |  | K | DAMAGING | 0 | *Warning! Low confidence.** | DAMAGING | 0 | *Warning! Low confidence.** |
| rs2228570 | M1R | NP_000367 | M | TOLERATED | 1 | 4.32 | TOLERATED | 1 | 4.32 |
|  |  |  | R | DAMAGING | 0 | *Warning! Low confidence.** | DAMAGING | 0 | *Warning! Low confidence.** |
| rs2228570 | M1T | NP_000367 | M | TOLERATED | 1 | 4.32 | TOLERATED | 1 | 4.32 |
|  |  |  | T | DAMAGING | 0 | *Warning! Low confidence.** | DAMAGING | 0 | *Warning! Low confidence.** |
| rs2241880 | T216A | NP_001177195 | T | TOLERATED | 0.61 | 1.9 | TOLERATED | 1 | 3.47 |
|  |  |  | A | TOLERATED | 0.79 |  | TOLERATED | 0.42 |  |
| rs231775 | T17A | NP_001032720 | T | TOLERATED | 1 | 3.32 | TOLERATED | 0.43 | 3.92 |
|  |  |  | A | TOLERATED | 0.09 |  | TOLERATED | 0.13 |  |
| rs28371759 | L293P | NP_059488 | L | TOLERATED | 0.32 | 1.96 | TOLERATED | 1 | 4.32 |
|  |  |  | P | TOLERATED | 0.22 |  | DAMAGING | 0 | *Warning! Low confidence.** |
| rs3135932 | S159G | NP_001549 | S | TOLERATED | 0.92 | 2.43 | TOLERATED | 1 | 2.53 |
|  |  |  | G | TOLERATED | 0.38 |  | TOLERATED | 0.29 |  |
| rs34069356 | A286T | NP_001327 | A | TOLERATED | 0.89 | 2.55 | TOLERATED | 0.87 | 3.94 |
|  |  |  | T | TOLERATED | 0.45 |  | TOLERATED | 0.08 |  |
| rs3734114 | S62P | NP_001124500 | S | TOLERATED | 1 | 1.6 | TOLERATED | 1 | 2.12 |
|  |  |  | P | TOLERATED | 0.26 |  | TOLERATED | 0.1 |  |
| rs3751143 | E496A | NP_002553 | E | TOLERATED | 1 | 3.28 | TOLERATED | 1 | 3.3 |
|  |  |  | A | DAMAGING | 0.01 | *Warning! Low confidence.** | DAMAGING | 0.01 | *Warning! Low confidence.** |
| rs375947 | M365T | NP_005526 | M | TOLERATED | 0.17 | 2.33 | TOLERATED | 0.18 | 2.17 |
|  |  |  | T | TOLERATED | 0.66 |  | TOLERATED | 0.41 |  |
| rs3764880 | M1V | NP_619542 | M | TOLERATED | 1 | 4.32 | TOLERATED | 1 | 3.55 |
|  |  |  | V | DAMAGING | 0 | *Warning! Low confidence.** | DAMAGING | 0 | *Warning! Low confidence.** |
| rs3827103 | V44I | NP_063941 | V | TOLERATED | 1 | 3.47 | TOLERATED | 1 | 3.9 |
|  |  |  | I | DAMAGING | 0.05 | *Warning! Low confidence.** | TOLERATED | 0.06 |  |
| rs401502 | G378R | NP_005526 | G | TOLERATED | 0.76 | 2.45 | TOLERATED | 0.84 | 2.2 |
|  |  |  | R | TOLERATED | 0.63 |  | TOLERATED | 0.62 |  |
| rs482912 | I318V | NP_055213 | I | TOLERATED | 0.45 | 2.3 | TOLERATED | 0.94 | 2.35 |
|  |  |  | V | TOLERATED | 0.6 |  | TOLERATED | 1 |  |
| rs4833095 | N248S | NP_003254 | N | TOLERATED | 1 | 1.86 | TOLERATED | 1 | 4.08 |
|  |  |  | S | TOLERATED | 0.5 |  | TOLERATED | 0.93 |  |
| rs4986790 | D299G | NP_612564 | D | TOLERATED | 1 | 1.97 | TOLERATED | 1 | 4.09 |
|  |  |  | G | TOLERATED | 0.15 |  | DAMAGING | 0.01 | *Warning! Low confidence.** |
| rs5030737 | R52C | NP_000233 | R | TOLERATED | 1 | 1.87 | TOLERATED | 1 | 3.5 |
|  |  |  | C | DAMAGING | 0.02 |  | DAMAGING | 0 | *Warning! Low confidence.** |
| rs5743278 | A725G | NP_071445 | A | TOLERATED | 1 | 2.44 | TOLERATED | 1 | 3.52 |
|  |  |  | G | TOLERATED | 0.2 |  | DAMAGING | 0.03 | *Warning! Low confidence.** |
| rs5743708 | R753Q | NP_003255 | R | TOLERATED | 1 | 1.9 | TOLERATED | 1 | 4.32 |
|  |  |  | Q | DAMAGING | 0.01 |  | DAMAGING | 0 | *Warning! Low confidence.** |
| rs61733329 | G749S | NP_775952 | G | TOLERATED | 0.74 | 2.3 | TOLERATED | 0.54 | 3.24 |
|  |  |  | S | TOLERATED | 0.97 |  | TOLERATED | 0.51 |  |
| rs71497223 | T399A | NP_002429 | T | TOLERATED | 0.27 | 1.78 | TOLERATED | 0.31 | 2.44 |
|  |  |  | A | TOLERATED | 0.99 |  | TOLERATED | 1 |  |
| rs72553867 | T94K | NP_001139277 | T | TOLERATED | 0.41 | 2.72 | TOLERATED | 0.2 | 3.01 |
|  |  |  | K | TOLERATED | 0.06 |  | DAMAGING | 0.04 |  |
| rs74719094 | R1701S | NP_060506 | R | TOLERATED | 1 | 1.77 | TOLERATED | 1 | 2.42 |
|  |  |  | S | DAMAGING | 0.01 |  | DAMAGING | 0 |  |
| rs77228473 | E1499D | NP_055919 | E | TOLERATED | 1 | 2.07 | TOLERATED | 1 | 2.42 |
|  |  |  | D | TOLERATED | 0.82 |  | TOLERATED | 0.8 |  |
| rs77833427 | R566H | NP_055919 | R | TOLERATED | 0.57 | 2.06 | TOLERATED | 1 | 2.44 |
|  |  |  | H | TOLERATED | 0.14 |  | TOLERATED | 0.21 |  |
| rs8177374 | S180L | NP_001034750 | S | TOLERATED | 0.87 | 2.28 | TOLERATED | 0.61 | 2.7 |
|  |  |  | L | TOLERATED | 0.69 |  | TOLERATED | 0.73 |  |
| rs8177400 | D96N | NP_001034750 | D | TOLERATED | 1 | 2.21 | TOLERATED | 1 | 2.5 |
|  |  |  | N | DAMAGING | 0.03 |  | DAMAGING | 0 |  |
| rs883541 | T31I | NP_060453 | T | DAMAGING | 0.01 | 2.68 | DAMAGING | 0.02 | 3.72 |
|  |  |  | I | TOLERATED | 0.18 |  | TOLERATED | 0.81 | *Warning! Low confidence.** |
| rs9061 | E213K | NP_001171944 | E | TOLERATED | 1 | 2.06 | TOLERATED | 1 | 2.43 |
|  |  |  | K | TOLERATED | 0.89 |  | TOLERATED | 0.91 |  |
| rs9273665 | A222D | NP_002114 | A | TOLERATED | 1 | 2.88 | TOLERATED | 1 | 4.32 |
|  |  |  | D | DAMAGING | 0.02 |  | DAMAGING | 0 | *Warning! Low confidence.** |
| rs9323945 | N1124D | NP_060506 | N | TOLERATED | 0.58 | 2.03 | TOLERATED | 0.31 | 2.4 |
|  |  |  | D | TOLERATED | 1 |  | TOLERATED | 1 |  |
| rs9577229 | A204V | NP_005552 | A | TOLERATED | 0.24 | 1.84 | TOLERATED | 0.56 | 2.85 |
|  |  |  | V | TOLERATED | 1 |  | TOLERATED | 1 |  |
| rs9943208 | G354R | NP_003456 | G | TOLERATED | 1 | 2.23 | TOLERATED | 1 | 3.32 |
|  |  |  | R | DAMAGING | 0 |  | DAMAGING | 0 | *Warning! Low confidence.** |
| rs2229094 | C13R | NP_000586 | C | TOLERATED | 0.11 | 2.9 | DAMAGING | 0.04 | 4.32 |
|  |  |  | R | TOLERATED | 0.22 |  | TOLERATED | 0.85 | *Warning! Low confidence.** |
| rs5743810 | S249P | NP_006059 | S | TOLERATED | 0.51 | 1.93 | DAMAGING | 0.02 | 4.18 |
|  |  |  | P | TOLERATED | 0.25 |  | TOLERATED | 1 | *Warning! Low confidence.** |
| rs6761637 | F282S | NP_006761 | F | TOLERATED | 0.07 | 2.2 | TOLERATED | 0.13 | 2.99 |
|  |  |  | S | TOLERATED | 0.9 |  | TOLERATED | 0.83 |  |
| rs11556887 | A134V | NP_001171944 | A | TOLERATED | 1 | 2.11 | TOLERATED | 1 | 2.4 |
|  |  |  | V | TOLERATED | 0.17 |  | TOLERATED | 0.26 |  |
| rs3802813 | S55N | NP_001034750 | S | TOLERATED | 1 | 2.52 | TOLERATED | 1 | 2.71 |
|  |  |  | N | TOLERATED | 0.24 |  | TOLERATED | 0.48 |  |
| rs2076530 | S360G | NP_062548 | S | TOLERATED | 0.45 | 1.43 | TOLERATED | 0.28 | 3.26 |
