# Supplementary material for: HGV&TB: a comprehensive online resource on human genes and genetic variants associated with tuberculosis
Source: Database (Oxford). 2014 Dec 13;2014:bau112. doi: 10.1093/database/bau112 (PMC5630898; doi:10.1093/database/bau112)
Supplement: Supplementary Data [file bau112_Supplementary_Data.zip › Supplementary_Table_7.docx]

**Supplementary Table 7: Allele frequencies of variations from the HapMap project**

|  | ASW | CEU | CHB | CHD | GIH | JPT | LWK | MEX | MKK | TSI | YRI |
| --- | --- | --- | --- | --- | --- | --- | --- | --- | --- | --- | --- |
| rs165172 |  | 0.25 | 0.422 |  |  | 0.389 |  |  |  |  | 0.535 |
| rs118823 | 0.283 | 0.4 | 0.6 |  | 0.4 |  | 0.25 | 0.4 | 0.21 | 0.17 | 0.35 |
| rs118868 | 0.283 | 0.425 | 0.233 |  | 0.4 | 0.148 | 0.25 | 0.4 | 0.21 | 0.17 | 0.567 |
| rs12229 | 0.274 | 0.45 | 0.18 | 0.47 | 0.46 | 0.29 | 0.267 | 0.51 | 0.189 | 0.8 | 0.35 |
| rs12941 | 0.274 | 0.392 | 0.467 | 0.47 | 0.46 | 0.49 | 0.267 | 0.51 | 0.189 | 0.8 | 0.175 |
| rs1245298 | 0.821 | 0.93 | 0.946 | 0.935 | 0.937 | 0.942 | 0.867 | 0.89 | 0.895 | 0.99 | 0.872 |
| rs124611 | 0.245 | 0.35 | 0.625 | 0.565 | 0.256 | 0.628 | 0.267 | 0.46 | 0.23 | 0.29 | 0.24 |
| rs1369 | 0.283 | 0.265 | 0.131 | 0.26 | 0.33 | 0.81 | 0.211 | 0.22 | 0.318 | 0.284 | 0.164 |
| rs141981 | 0.5 | 0.336 | 0.435 | 0.465 | 0.239 | 0.41 | 0.578 | 0.28 | 0.584 | 0.293 | 0.483 |
| rs149111 | 0.5 | 0.75 | 0.127 | 0.118 | 0.227 | 0.134 | 0.344 | 0.9 | 0.343 | 0.91 | 0.483 |
| rs1492975 | 0.5 |  | 0.89 | 0.118 | 0.227 | 0.45 | 0.344 | 0.9 | 0.343 | 0.91 | 0.175 |
| rs1493327 | 0.49 | 0.395 | 0.627 | 0.665 | 0.347 | 0.676 | 0.478 | 0.531 | 0.538 | 0.455 | 0.535 |
| rs1493328 | 0.179 | 0.159 | 0.42 | 0.24 | 0.25 | 0.6 | 0.15 | 0.19 | 0.168 | 0.159 | 0.12 |
| rs1497744 | 0.16 | 0.332 | 0.417 | 0.418 | 0.385 | 0.442 | 0.172 | 0.44 | 0.26 | 0.347 | 0.15 |
| rs1514611 | 0.217 | 0.263 | 0.214 | 0.2 | 0.18 | 0.276 | 0.272 | 0.23 | 0.168 | 0.165 | 0.21 |
| rs1515787 | 0.9 | 0.4 | 0.393 | 0.394 | 0.62 | 0.326 | 0.22 | 0.17 | 0.42 | 0.45 | 0.9 |
| rs159293 | 0.792 | 0.478 | 0.845 | 0.863 | 0.585 | 0.747 | 0.86 | 0.63 | 0.717 | 0.42 | 0.889 |
| rs161624 | 0.292 | 0.477 | 0.572 | 0.56 | 0.46 | 0.535 | 0.239 | 0.63 | 0.196 | 0.446 | 0.332 |
| rs165761 | 0.292 | 0.13 | 0.89 | 0.56 | 0.46 | 0.12 | 0.239 | 0.63 | 0.196 | 0.446 | 0.76 |
| rs173581 | 0.82 | 0.588 | 0.595 | 0.518 | 0.75 | 0.674 | 0.849 | 0.48 | 0.796 | 0.619 | 0.88 |
| rs192158 | 0.33 | 0.15 | 0.565 | 0.553 | 0.415 | 0.65 | 0.356 | 0.281 | 0.381 | 0.25 | 0.442 |
| rs11117415 | 0.226 | 0.53 | 0.196 | 0.26 | 0.23 | 0.279 | 0.294 | 0.8 | 0.325 | 0.4 | 0.358 |
| rs112926 | 0.19 | 0.41 |  | 0.6 | 0.23 |  | 0.6 | 0.71 | 0.31 | 0.91 | 0.9 |
| rs1135216 | 0.236 | 0.111 | 0.22 | 0.141 | 0.193 | 0.134 | 0.317 | 0.23 | 0.222 | 0.25 | 0.265 |
| rs1135791 | 0.349 | 0.513 | 0.19 | 0.153 | 0.477 | 0.163 | 0.256 | 0.32 | 0.371 | 0.54 | 0.142 |
| rs1143634 | 0.14 | 0.28 | 0.24 | 0.18 | 0.182 | 0.59 | 0.128 | 0.12 | 0.189 | 0.239 | 0.93 |
| rs11556887 | 0.19 | 0.84 | 0.71 | 0.94 | 0.253 | 0.6 | 0.17 | 0.2 | 0.8 | 0.142 |  |
| rs11568344 | 0.132 |  |  | 0.94 | 0.253 |  | 0.167 | 0.2 | 0.8 | 0.142 | 0.115 |
| rs1156835 | 0.85 |  |  | 0.94 | 0.253 |  | 0.23 | 0.11 | 0.1 | 0.142 | 0.45 |
| rs1156882 | 0.717 | 0.24 | 0.452 | 0.371 | 0.49 | 0.465 | 0.878 | 0.19 | 0.776 | 0.241 | 0.978 |
| rs11571319 | 0.717 | 0.26 |  | 0.371 | 0.49 |  | 0.878 | 0.19 | 0.776 | 0.241 |  |
| rs1157479 | 0.236 | 0.16 | 0.48 | 0.53 | 0.51 | 0.41 | 0.26 | 0.19 | 0.252 | 0.182 | 0.239 |
| rs11679983 | 0.236 | 0.15 | 0.23 | 0.53 | 0.51 | 0.11 | 0.26 | 0.19 | 0.252 | 0.182 | 0.44 |
| rs11884632 | 0.236 | 0.297 | 0.148 | 0.53 | 0.51 | 0.44 | 0.26 | 0.19 | 0.252 | 0.182 | 0.17 |
| rs1312833 | 0.236 | 0.567 | 0.58 | 0.53 | 0.51 | 0.7 | 0.26 | 0.19 | 0.252 | 0.182 | 0.27 |
| rs1327474 | 0.896 | 0.62 | 0.929 | 0.935 | 0.753 | 0.977 | 0.989 | 0.66 | 0.941 | 0.625 | 0.969 |
| rs1336436 | 0.896 |  | 0.44 | 0.935 | 0.753 | 0.44 | 0.989 | 0.66 | 0.941 | 0.625 |  |
| rs133896 | 0.28 | 0.111 | 0.95 | 0.82 | 0.68 | 0.93 | 0.26 | 0.11 | 0.171 | 0.18 | 0.217 |
| rs1371562 | 0.151 | 0.389 | 0.133 | 0.182 | 0.25 | 0.174 | 0.128 | 0.32 | 0.162 | 0.466 | 0.129 |
| rs1399431 | 0.151 | 0.475 | 0.233 | 0.182 | 0.25 | 0.279 | 0.128 | 0.32 | 0.162 | 0.466 | 0.38 |
| rs1418267 | 0.151 | 0.525 | 0.922 | 0.182 | 0.25 | 0.932 | 0.128 | 0.32 | 0.162 | 0.466 | 0.883 |
| rs1439814 | 0.358 | 0.62 | 0.827 | 0.835 | 0.62 | 0.872 | 0.183 | 0.71 | 0.343 | 0.62 | 0.296 |
| rs1439816 | 0.349 | 0.832 | 0.815 | 0.847 | 0.778 | 0.884 | 0.172 | 0.81 | 0.42 | 0.818 | 0.265 |
| rs1518111 | 0.613 | 0.788 | 0.256 | 0.318 | 0.619 | 0.366 | 0.6 | 0.62 | 0.629 | 0.744 | 0.527 |
| rs154441 | 0.217 | 0.438 | 0.42 | 0.41 | 0.42 | 0.122 | 0.25 | 0.24 | 0.369 | 0.414 | 0.279 |
| rs1552481 | 0.764 | 1 | 1 | 0.41 | 0.42 | 1 | 0.8 | 0.99 | 0.818 | 0.414 | 0.77 |
| rs1554286 | 0.623 | 0.827 | 0.268 | 0.324 | 0.642 | 0.372 | 0.6 | 0.64 | 0.629 | 0.81 | 0.531 |
| rs1568351 | 0.623 | 0.827 | 0.268 | 0.324 | 0.642 | 0.372 | 0.6 | 0.64 | 0.629 | 0.81 | 0.232 |
| rs15929 | 0.283 | 0.482 | 0.554 | 0.541 | 0.49 | 0.57 | 0.4 | 0.59 | 0.38 | 0.449 | 0.261 |
| rs159291 | 0.283 | 0.487 | 0.554 | 0.548 | 0.425 | 0.564 | 0.392 | 0.6 | 0.342 | 0.449 | 0.261 |
| rs159294 | 0.491 | 0.867 | 0.792 | 0.747 | 0.824 | 0.814 | 0.361 | 0.79 | 0.451 | 0.847 | 0.376 |
| rs159319 | 0.283 | 0.459 | 0.548 | 0.547 | 0.358 | 0.57 | 0.322 | 0.552 | 0.229 | 0.438 | 0.195 |
| rs16379 | 0.736 | 0.757 | 0.911 | 0.96 | 0.835 | 0.936 | 0.589 | 0.76 | 0.769 | 0.716 | 0.677 |
| rs1638 | 0.951 | 0.84 | 0.881 | 0.9 | 0.81 | 0.91 | 0.933 | 0.8 | 0.881 | 0.75 | 0.96 |
| rs164637 | 0.28 | 0.31 | 0.24 | 0.35 | 0.28 | 0.17 | 0.933 | 0.6 | 0.881 | 0.74 |  |
| rs1653624 | 0.28 | 0.983 | 1 | 0.35 | 0.28 | 1 | 0.933 | 0.6 | 0.881 | 0.74 | 1 |
| rs179726 | 0.28 | 0.9 | 0.128 | 0.35 | 0.28 | 0.51 | 0.933 | 0.6 | 0.881 | 0.74 | 1 |
| rs1723549 | 0.58 | 0.14 | 0.167 | 0.125 | 0.28 | 0.89 | 0.78 | 0.16 | 0.75 | 0.23 | 0.76 |
| rs17795618 | 0.142 | 0.159 | 0.28 | 0.188 | 0.244 | 0.273 | 0.94 | 0.16 | 0.127 | 0.136 | 0.115 |
| rs1845 | 0.38 | 0.15 | 0.155 | 0.165 | 0.142 | 0.174 | 0.28 | 0.15 | 0.42 | 0.125 | 0.9 |
| rs18451 | 0.189 | 0.18 | 0.6 | 0.165 | 0.6 | 0.6 | 0.146 | 0.31 | 0.15 | 0.125 | 0.252 |
| rs18587 | 0.337 | 0.252 | 0.6 | 0.71 | 0.295 | 0.128 | 0.35 | 0.25 | 0.345 | 0.267 | 0.46 |
| rs18629 | 0.57 | 0.173 | 0.89 | 0.41 | 0.62 | 0.23 | 0.94 | 0.6 | 0.8 | 0.97 | 0.88 |
| rs18795 | 0.896 | 0.465 | 1 | 0.994 | 0.875 | 1 | 0.94 | 0.84 | 0.951 | 0.648 | 1 |
| rs18796 | 0.896 | 0.43 | 0.767 | 0.994 | 0.875 | 0.864 | 0.94 | 0.84 | 0.951 | 0.648 | 0.93 |
| rs18797 | 0.96 | 0.473 | 1 | 0.994 | 0.892 | 1 | 0.94 | 0.85 | 0.951 | 0.648 | 1 |
| rs18871 | 0.96 | 0.827 | 0.256 | 0.994 | 0.892 | 0.312 | 0.94 | 0.85 | 0.951 | 0.648 | 0.536 |
| rs18872 | 0.613 | 0.788 | 0.25 | 0.318 | 0.619 | 0.366 | 0.6 | 0.622 | 0.629 | 0.722 | 0.527 |
| rs18896 | 0.34 | 0.531 | 0.36 | 0.59 | 0.284 | 0.52 | 0.372 | 0.32 | 0.416 | 0.432 | 0.274 |
| rs181157 | 0.77 | 0.28 | 0.298 | 0.282 | 0.312 | 0.291 | 0.51 | 0.15 | 0.84 | 0.29 | 0.22 |
| rs181672 | 0.78 | 0.862 | 0.994 | 0.282 | 0.93 | 1 | 0.562 | 0.86 | 0.671 | 0.869 | 0.5 |
| rs1861493 | 0.78 | 0.683 | 0.667 | 0.282 | 0.93 | 0.5 | 0.562 | 0.86 | 0.671 | 0.869 | 0.958 |
| rs1861494 | 0.78 | 0.664 | 0.667 | 0.282 | 0.93 | 0.511 | 0.562 | 0.86 | 0.671 | 0.869 | 0.898 |
| rs1864183 | 0.236 | 0.491 | 0.917 | 0.859 | 0.494 | 0.959 | 0.2 | 0.59 | 0.346 | 0.517 | 0.133 |
| rs1946518 | 0.632 | 0.68 | 0.39 | 0.464 | 0.756 | 0.424 | 0.646 | 0.54 | 0.656 | 0.511 | 0.655 |
| rs211839 | 0.85 | 0.381 | 0.54 | 0.53 | 0.114 | 0.93 | 0.61 | 0.34 | 0.8 | 0.494 | 0.31 |
| rs23192 | 0.85 | 0.62 | 0.265 | 0.2 | 0.11 | 0.192 | 0.61 | 0.13 | 0.8 | 0.51 |  |
| rs257178 | 0.85 | 0.125 | 0.23 | 0.2 | 0.11 | 0.45 | 0.61 | 0.13 | 0.8 | 0.51 | 0.333 |
| rs266842 | 0.123 | 0.341 |  | 0.12 | 0.85 | 0.6 | 0.17 | 0.194 | 0.92 | 0.239 | 0.9 |
| rs266844 | 0.9 | 0.341 |  | 0.6 | 0.18 | 0.6 | 0.11 | 0.4 | 0.4 | 0.239 | 0.9 |
| rs269824 | 0.132 | 0.4 | 0.6 | 0.6 | 0.136 |  | 0.156 | 0.8 | 0.115 | 0.153 | 0.111 |
| rs27874 | 0.368 | 0.137 | 0.738 | 0.794 | 0.125 | 0.721 | 0.583 | 0.42 | 0.329 | 0.142 | 0.473 |
| rs271543 | 0.113 | 0.168 | 0.161 | 0.218 | 0.25 | 0.163 | 0.44 | 0.23 | 0.185 | 0.159 | 0.8 |
| rs27269 | 0.113 | 0.517 | 0.444 | 0.218 | 0.25 | 0.398 | 0.44 | 0.23 | 0.185 | 0.159 | 0.319 |
| rs27653 | 0.321 | 0.469 | 0.331 | 0.179 | 0.47 | 0.488 | 0.194 | 0.57 | 0.189 | 0.368 | 0.292 |
| rs217538 | 0.396 | 0.155 | 0.349 | 0.282 | 0.295 | 0.341 | 0.483 | 0.312 | 0.42 | 0.178 | 0.425 |
| rs21837 | 0.745 | 0.916 | 0.81 | 0.765 | 0.87 | 0.843 | 0.672 | 0.89 | 0.713 | 0.881 | 0.58 |
| rs2114592 | 0.113 | 0.94 | 0.259 | 0.182 | 0.261 | 0.134 | 0.289 | 0.2 | 0.359 | 0.161 | 0.173 |
| rs222865 | 0.123 |  | 0.12 | 0.18 | 0.6 | 0.35 | 0.94 | 0.2 | 0.28 | 0.161 | 0.122 |
| rs222994 | 0.33 | 0.252 | 0.214 | 0.212 | 0.386 | 0.151 | 0.278 | 0.3 | 0.248 | 0.33 | 0.283 |
| rs2229238 | 0.679 | 0.783 | 0.875 | 0.835 | 0.795 | 0.831 | 0.817 | 0.82 | 0.832 | 0.828 | 0.841 |
| rs2234711 | 0.679 | 0.35 | 0.5 | 0.835 | 0.795 | 0.442 | 0.817 | 0.82 | 0.832 | 0.828 | 0.469 |
| rs224188 | 0.274 | 0.567 | 0.387 | 0.238 | 0.494 | 0.29 | 0.213 | 0.3 | 0.28 | 0.535 | 0.27 |
| rs224325 | 0.557 | 0.137 | 0.738 | 0.794 | 0.114 | 0.724 | 0.817 | 0.429 | 0.535 | 0.136 | 0.788 |
| rs2245214 | 0.557 | 0.432 | 0.533 | 0.794 | 0.114 | 0.489 | 0.817 | 0.429 | 0.535 | 0.136 | 0.45 |
| rs2248814 | 0.557 | 0.517 | 0.678 | 0.794 | 0.114 | 0.682 | 0.817 | 0.429 | 0.535 | 0.136 | 0.925 |
| rs2255929 | 0.557 | 0.339 | 0.678 | 0.794 | 0.114 | 0.67 | 0.817 | 0.429 | 0.535 | 0.136 | 0.675 |
| rs227361 | 0.481 | 0.367 | 0.56 | 0.659 | 0.477 | 0.587 | 0.539 | 0.68 | 0.78 | 0.489 | 0.434 |
| rs2274894 | 0.868 | 0.562 | 0.714 | 0.673 | 0.841 | 0.715 | 0.844 | 0.61 | 0.899 | 0.585 | 0.898 |
| rs2276631 | 0.16 | 0.216 | 0.137 | 0.113 | 0.91 | 0.112 | 0.144 | 0.281 | 0.282 | 0.25 | 0.152 |
| rs2278588 | 0.16 | 0.2 | 0.256 | 0.113 | 0.91 | 0.318 | 0.144 | 0.281 | 0.282 | 0.25 | 0.1 |
| rs228788 | 0.16 | 0.9 | 0.149 | 0.113 | 0.91 | 0.176 | 0.144 | 0.281 | 0.282 | 0.25 |  |
| rs228789 | 0.236 | 0.12 | 0.321 | 0.276 | 0.119 | 0.331 | 0.228 | 0.27 | 0.14 | 0.136 | 0.173 |
| rs23474 | 0.179 | 0.619 | 0.786 | 0.788 | 0.653 | 0.86 | 0.139 | 0.76 | 0.311 | 0.642 | 0.195 |
| rs231489 | 0.66 | 0.549 | 0.72 | 0.676 | 0.835 | 0.79 | 0.66 | 0.5 | 0.748 | 0.58 | 0.624 |
| rs231775 | 0.387 | 0.389 | 0.696 | 0.673 | 0.29 | 0.635 | 0.489 | 0.378 | 0.49 | 0.29 | 0.363 |
| rs2352262 | 0.689 | 0.628 | 0.892 | 0.876 | 0.722 | 0.91 | 0.594 | 0.8 | 0.727 | 0.665 | 0.637 |
| rs247661 | 0.98 | 0.883 | 0.982 | 0.988 | 0.989 | 0.982 | 0.994 | 0.948 | 0.997 | 0.93 | 0.995 |
| rs274553 | 0.98 | 0.828 | 0.867 | 0.988 | 0.989 | 0.966 | 0.994 | 0.948 | 0.997 | 0.93 | 0.458 |
| rs274554 | 0.519 | 0.819 | 0.875 | 0.888 | 0.824 | 0.971 | 0.389 | 0.86 | 0.413 | 0.835 | 0.425 |
| rs274559 | 0.34 | 0.385 | 0.577 | 0.559 | 0.665 | 0.738 | 0.261 | 0.35 | 0.318 | 0.49 | 0.35 |
| rs2834213 | 0.47 | 0.217 | 0.155 | 0.135 | 0.193 | 0.23 | 0.5 | 0.22 | 0.49 | 0.278 | 0.9 |
| rs28371759 | 0.47 | 0.217 | 0.155 | 0.18 | 0.193 | 0.17 | 0.5 | 0.22 | 0.49 | 0.278 | 0.9 |
| rs2839693 | 0.47 | 0.99 | 0.936 | 0.18 | 0.193 | 0.988 | 0.5 | 0.22 | 0.49 | 0.278 | 0.783 |
| rs2853694 | 0.755 | 0.456 | 0.673 | 0.694 | 0.477 | 0.75 | 0.822 | 0.7 | 0.811 | 0.62 | 0.792 |
| rs387243 | 0.226 | 0.46 | 0.196 | 0.235 | 0.653 | 0.273 | 0.117 | 0.39 | 0.147 | 0.523 | 0.181 |
| rs391324 | 0.85 | 0.164 | 0.143 | 0.182 | 0.114 | 0.147 | 0.117 | 0.15 | 0.129 | 0.159 | 0.71 |
| rs3135932 | 0.38 | 0.221 |  | 0.182 | 0.195 |  | 0.117 | 0.5 | 0.129 | 0.131 |  |
| rs313831 | 0.28 | 0.183 | 0.12 | 0.18 | 0.176 | 0.23 | 0.6 | 0.13 | 0.21 | 0.222 | 0.4 |
| rs321222 | 0.28 | 0.225 | 0.411 | 0.18 | 0.176 | 0.489 | 0.6 | 0.13 | 0.21 | 0.222 | 0.3 |
| rs3212227 | 0.321 | 0.19 | 0.423 | 0.429 | 0.335 | 0.558 | 0.428 | 0.37 | 0.294 | 0.239 | 0.327 |
| rs352139 | 0.64 | 0.482 | 0.45 | 0.387 | 0.455 | 0.56 | 0.55 | 0.59 | 0.566 | 0.693 | 0.575 |
| rs352143 | 0.46 | 0.186 | 0.3 | 0.41 | 0.114 | 0.41 | 0.372 | 0.16 | 0.46 | 0.278 | 0.385 |
| rs372958 | 0.865 | 0.544 | 0.554 | 0.482 | 0.792 | 0.471 | 0.85 | 0.75 | 0.876 | 0.557 | 0.863 |
| rs3729718 | 0.85 |  |  | 0.482 | 0.792 |  | 0.144 | 0.75 | 0.28 | 0.557 | 0.97 |
| rs3731863 | 0.142 | 0.53 | 0.113 | 0.88 | 0.91 | 0.76 | 0.133 | 0.7 | 0.52 | 0.62 | 0.146 |
| rs3734114 | 0.57 | 0.174 | 0.199 | 0.235 | 0.384 | 0.93 | 0.89 | 0.2 | 0.94 | 0.21 | 0.66 |
| rs37592 | 0.236 | 0.46 | 0.292 | 0.288 | 0.369 | 0.273 | 0.278 | 0.44 | 0.336 | 0.483 | 0.332 |
| rs3751143 | 0.66 | 0.177 | 0.235 | 0.271 | 0.261 | 0.267 | 0.1 | 0.224 | 0.189 | 0.278 | 0.93 |
| rs375947 | 0.255 | 0.353 | 0.292 | 0.387 | 0.227 | 0.382 | 0.222 | 0.14 | 0.294 | 0.385 | 0.257 |
| rs3761624 | 0.255 | 0.222 | 0.769 | 0.387 | 0.227 | 0.727 | 0.222 | 0.14 | 0.294 | 0.385 | 0.278 |
| rs3763313 | 0.226 | 0.212 | 0.28 | 0.218 | 0.182 | 0.198 | 0.244 | 0.21 | 0.38 | 0.199 | 0.181 |
| rs3763317 | 0.538 | 0.54 | 0.595 | 0.595 | 0.648 | 0.79 | 0.55 | 0.54 | 0.549 | 0.448 | 0.518 |
| rs3764879 | 0.538 | 0.211 | 0.765 | 0.595 | 0.648 | 0.727 | 0.55 | 0.54 | 0.549 | 0.448 | 0.311 |
| rs376488 | 0.259 | 0.249 | 0.786 | 0.868 | 0.527 | 0.766 | 0.348 | 0.453 | 0.358 | 0.326 | 0.286 |
| rs3788935 | 0.212 | 0.249 | 0.786 | 0.867 | 0.54 | 0.766 | 0.341 | 0.453 | 0.356 | 0.326 | 0.268 |
| rs379279 | 0.321 |  | 0.155 | 0.135 | 0.6 | 0.7 | 0.25 | 0.4 | 0.157 | 0.23 | 0.179 |
| rs382813 | 0.28 | 0.4 | 0.125 | 0.155 | 0.8 | 0.163 | 0.16 | 0.2 | 0.35 | 0.57 | 0.4 |
| rs382814 | 0.57 | 0.15 | 0.6 | 0.24 | 0.136 | 0.29 | 0.16 | 0.8 | 0.35 | 0.176 | 0.4 |
| rs38499 | 0.615 | 0.451 | 0.339 | 0.324 | 0.295 | 0.267 | 0.7 | 0.35 | 0.636 | 0.432 | 0.628 |
| rs3841 | 0.57 | 0.75 | 0.321 | 0.34 | 0.142 | 0.217 | 0.62 | 0.9 | 0.24 | 0.62 | 0.62 |
| rs3811621 | 0.57 | 0.725 | 0.867 | 0.34 | 0.142 | 0.852 | 0.62 | 0.9 | 0.24 | 0.62 | 0.68 |
| rs382713 | 0.46 | 0.8 | 0.226 | 0.229 | 0.199 | 0.227 | 0.372 | 0.13 | 0.437 | 0.85 | 0.558 |
| rs388714 | 0.132 | 0.162 | 0.114 | 0.65 | 0.176 | 0.227 | 0.152 | 0.214 | 0.85 | 0.182 | 0.558 |
| rs3948464 | 0.877 | 0.854 | 0.994 | 0.65 | 0.949 | 1 | 0.772 | 0.94 | 0.752 | 0.915 | 0.876 |
| rs4152 | 0.877 | 0.375 | 0.398 | 0.65 | 0.949 | 0.386 | 0.772 | 0.94 | 0.752 | 0.915 | 0.186 |
| rs4149578 | 0.226 | 0.93 | 0.298 | 0.241 | 0.256 | 0.18 | 0.25 | 0.12 | 0.224 | 0.74 | 0.31 |
| rs4149622 | 0.226 |  | 0.1 | 0.241 | 0.256 | 0.125 | 0.25 | 0.12 | 0.224 | 0.74 | 0.65 |
| rs4149623 | 0.226 |  | 0.1 | 0.241 | 0.256 | 0.122 | 0.25 | 0.12 | 0.224 | 0.74 | 0.653 |
| rs4149639 | 0.226 |  | 0.114 | 0.241 | 0.256 | 0.68 | 0.25 | 0.12 | 0.224 | 0.74 | 0.22 |
| rs4244285 | 0.226 | 0.155 | 0.256 | 0.241 | 0.256 | 0.284 | 0.25 | 0.12 | 0.224 | 0.74 | 0.144 |
| rs425219 | 0.226 | 0.127 | 0.533 | 0.241 | 0.256 | 0.622 | 0.25 | 0.12 | 0.224 | 0.74 | 0.592 |
| rs437967 | 0.226 | 0.758 | 0.889 | 0.241 | 0.256 | 0.811 | 0.25 | 0.12 | 0.224 | 0.74 | 0.88 |
| rs451635 | 0.14 | 0.381 | 0.12 | 0.24 | 0.176 | 0.6 | 0.39 | 0.22 | 0.112 | 0.438 | 0.4 |
| rs4586 | 0.575 | 0.358 | 0.625 | 0.565 | 0.398 | 0.629 | 0.683 | 0.54 | 0.622 | 0.358 | 0.761 |
| rs482912 | 0.349 | 0.642 | 0.524 | 0.529 | 0.659 | 0.378 | 0.36 | 0.49 | 0.399 | 0.71 | 0.177 |
| rs483395 | 0.792 | 0.212 | 0.669 | 0.631 | 0.472 | 0.7 | 0.922 | 0.45 | 0.839 | 0.49 | 0.884 |
| rs4896243 | 0.783 | 0.5 | 0.923 | 0.935 | 0.75 | 0.977 | 0.783 | 0.58 | 0.81 | 0.574 | 0.823 |
| rs4958846 | 0.783 | 0.5 | 0.75 | 0.935 | 0.75 | 0.533 | 0.783 | 0.58 | 0.81 | 0.574 | 0.552 |
| rs4958847 | 0.481 | 0.115 | 0.619 | 0.694 | 0.199 | 0.558 | 0.41 | 0.22 | 0.371 | 0.153 | 0.553 |
| rs496888 | 0.264 | 0.345 | 0.22 | 0.159 | 0.172 | 0.169 | 0.417 | 0.34 | 0.259 | 0.176 | 0.319 |
| rs498679 | 0.57 | 0.35 |  | 0.159 | 0.98 |  | 0.97 | 0.31 | 0.85 | 0.45 | 0.4 |
| rs53729 | 0.57 |  |  | 0.159 | 0.98 |  | 0.97 | 0.31 | 0.85 | 0.45 | 0.133 |
| rs53737 | 0.57 | 0.71 | 0.6 | 0.159 | 0.23 | 0.6 | 0.11 | 0.2 | 0.59 | 0.46 | 0.133 |
| rs5743278 | 0.57 |  |  | 0.159 | 0.23 |  | 0.11 | 0.2 | 0.59 | 0.46 | 0.83 |
| rs574378 | 0.57 | 0.52 |  | 0.159 | 0.23 |  | 0.11 | 0.2 | 0.59 | 0.46 |  |
| rs574381 | 0.915 | 0.536 | 1 | 0.159 | 0.977 | 1 | 0.989 | 0.81 | 0.96 | 0.665 | 1 |
| rs5743899 | 0.623 | 0.821 | 0.613 | 0.582 | 0.784 | 0.599 | 0.517 | 0.77 | 0.648 | 0.83 | 0.63 |
| rs6127698 | 0.14 | 0.5 | 0.667 | 0.659 | 0.528 | 0.698 | 0.144 | 0.571 | 0.122 | 0.43 | 0.62 |
| rs671524 | 0.59 | 0.237 | 0.83 | 0.124 | 0.148 | 0.11 | 0.544 | 0.163 | 0.437 | 0.273 | 0.571 |
| rs676281 | 0.717 | 0.765 | 0.744 | 0.81 | 0.659 | 0.82 | 0.522 | 0.82 | 0.65 | 0.767 | 0.597 |
| rs6761637 | 0.717 | 0.42 | 0.156 | 0.81 | 0.659 | 0.68 | 0.522 | 0.82 | 0.65 | 0.767 | 0.35 |
| rs6985962 | 0.717 | 0.117 | 0.78 | 0.81 | 0.659 | 0.12 | 0.522 | 0.82 | 0.65 | 0.767 | 0.144 |
| rs715848 | 0.717 | 0.153 | 0.616 | 0.81 | 0.659 | 0.58 | 0.522 | 0.82 | 0.65 | 0.767 | 0.683 |
| rs7111432 | 0.547 | 0.664 | 0.381 | 0.351 | 0.534 | 0.388 | 0.583 | 0.41 | 0.546 | 0.68 | 0.545 |
| rs7194886 | 0.274 | 0.372 | 0.17 | 0.143 | 0.233 | 0.15 | 0.22 | 0.23 | 0.395 | 0.472 | 0.23 |
| rs7215373 | 0.274 | 0.373 | 0.511 | 0.143 | 0.233 | 0.43 | 0.22 | 0.23 | 0.395 | 0.472 | 0.9 |
| rs722555 | 0.575 | 0.562 | 0.381 | 0.465 | 0.58 | 0.41 | 0.533 | 0.44 | 0.587 | 0.597 | 0.558 |
| rs7234985 | 0.575 | 0.275 | 0.447 | 0.465 | 0.58 | 0.427 | 0.533 | 0.44 | 0.587 | 0.597 | 0.558 |
| rs731236 | 0.236 | 0.438 | 0.36 | 0.41 | 0.295 | 0.118 | 0.272 | 0.26 | 0.434 | 0.414 | 0.288 |
| rs735239 | 0.57 | 0.376 | 0.171 | 0.173 | 0.273 | 0.194 | 0.11 | 0.41 | 0.52 | 0.32 | 0.4 |
| rs73524 | 0.245 | 0.451 | 0.28 | 0.235 | 0.369 | 0.267 | 0.422 | 0.44 | 0.34 | 0.432 | 0.327 |
| rs7573954 | 0.33 | 0.336 | 0.351 | 0.321 | 0.358 | 0.413 | 0.328 | 0.32 | 0.338 | 0.33 | 0.434 |
| rs767569 | 0.33 | 0.9 | 0.83 | 0.321 | 0.358 | 0.78 | 0.328 | 0.32 | 0.338 | 0.33 | 0.276 |
| rs774939 | 0.33 | 0.44 | 0.512 | 0.321 | 0.358 | 0.455 | 0.328 | 0.32 | 0.338 | 0.33 | 0.59 |
| rs776746 | 0.632 | 0.36 | 0.31 | 0.235 | 0.244 | 0.267 | 0.872 | 0.26 | 0.514 | 0.51 | 0.845 |
| rs7791836 | 0.442 | 0.576 | 0.45 | 0.333 | 0.517 | 0.47 | 0.567 | 0.38 | 0.5 | 0.545 | 0.544 |
| rs7932766 | 0.217 | 0.243 | 0.43 | 0.12 | 0.161 | 0.65 | 0.118 | 0.143 | 0.234 | 0.233 | 0.128 |
| rs7975232 | 0.632 | 0.571 | 0.31 | 0.3 | 0.517 | 0.343 | 0.733 | 0.45 | 0.675 | 0.597 | 0.628 |
| rs8177374 | 0.47 | 0.171 | 0.6 | 0.29 | 0.131 | 0.3 | 0.6 | 0.1 | 0.46 | 0.21 |  |
| rs81774 | 0.47 | 0.171 | 0.6 | 0.29 | 0.131 | 0.6 | 0.6 | 0.1 | 0.46 | 0.21 | 0.4 |
| rs883541 | 0.868 | 0.792 | 0.583 | 0.518 | 0.739 | 0.576 | 0.85 | 0.61 | 0.818 | 0.773 | 0.881 |
| rs961 | 0.868 | 0.792 | 0.25 | 0.518 | 0.739 | 0.286 | 0.85 | 0.61 | 0.818 | 0.773 | 0.881 |
| rs925994 | 0.163 | 0.37 | 0.185 | 0.173 | 0.47 | 0.253 | 0.194 | 0.61 | 0.73 | 0.52 | 0.117 |
| rs9268492 | 0.163 | 0.37 | 0.375 | 0.173 | 0.47 | 0.286 | 0.194 | 0.61 | 0.73 | 0.52 | 0.322 |
| rs9268494 | 0.33 | 0.363 | 0.44 | 0.418 | 0.511 | 0.512 | 0.272 | 0.38 | 0.22 | 0.284 | 0.398 |
| rs9282799 | 0.33 |  |  | 0.418 | 0.511 |  | 0.272 | 0.38 | 0.22 | 0.284 | 0.18 |
| rs9323945 | 0.991 | 0.987 | 0.762 | 0.753 | 0.818 | 0.82 | 0.989 | 0.38 | 0.989 | 0.994 | 1 |
| rs94598 | 0.14 | 0.18 | 0.214 | 0.182 | 0.148 | 0.256 | 0.39 | 0.4 | 0.84 | 0.34 | 0.66 |
| rs944722 | 0.14 | 0.58 | 0.7 | 0.182 | 0.148 | 0.682 | 0.39 | 0.4 | 0.84 | 0.34 | 0.98 |
| rs976 | 0.736 | 0.659 | 0.798 | 0.829 | 0.636 | 0.843 | 0.68 | 0.6 | 0.794 | 0.625 | 0.739 |
| rs994226 | 0.736 | 0.98 | 0.922 | 0.829 | 0.636 | 0.955 | 0.68 | 0.6 | 0.794 | 0.625 | 0.692 |
