# Supplementary material for: HGV&TB: a comprehensive online resource on human genes and genetic variants associated with tuberculosis
Source: Database (Oxford). 2014 Dec 13;2014:bau112. doi: 10.1093/database/bau112 (PMC5630898; doi:10.1093/database/bau112)
Supplement: Supplementary Data [file bau112_Supplementary_Data.zip › New_Microsoft_Office_Word_Document.docx]

**Supplementary Figure 1:** Intersection of variations predicted by SIFT and PolyPhen2

**Supplementary Figure 2:** Gene-gene interaction chart of 98 genes present in HGV&TB; obtained from STRING v.9.0.

**Supplementary Figure 3:** Frequency distribution of 202 SNPs whose frequency could be retrieved from HapMart across different populations

**Supplementary Table 1:** List of the entire Tuberculosis associated gene variant RSID’s associated with respective genes, having both individual and haplotype/diplotype/interaction association with Tuberculosis susceptibility; uploaded in HGV&TB.

**Supplementary Table 2:** List of all the Tuberculosis associated gene variants (whose RSID’s are not available), having both individual and haplotype/diplotype/interaction association with Tuberculosis susceptibility; uploaded in HGV&TB.

**Supplementary Table 3:** Classes of Diseases mapping to Genes involved in Genetic Susceptibility to tuberculosis

**Supplementary Table 4:** Counts of 307 associated variations in different genomic loci

**Supplementary Table 5:** Functional consequences of the variations as predicted by PolyPhen2 and SIFT

**Supplementary Table 6:** Population frequency distribution of TB susceptible genes present in HGV&TB

**Supplementary Table 7:** Allele frequencies of variations from the HapMap project

**Supplementary Table 8:** Variations showing evidence of selection
