# Supplementary material for: HGV&TB: a comprehensive online resource on human genes and genetic variants associated with tuberculosis
Source: Database (Oxford). 2014 Dec 13;2014:bau112. doi: 10.1093/database/bau112 (PMC5630898; doi:10.1093/database/bau112)
Supplement: Supplementary Data [file bau112_Supplementary_Data.zip › Supplementary_Table_8.docx]

**Supplementary Table 8:** Variations showing evidence of selection

|  | **RSID** | **Chromosome** | **Position** | **Integrated Haplotype Score (iHS)** | **Population** |
| --- | --- | --- | --- | --- | --- |
|  | rs11209 | 17 | 5230304 | -2.229452049 | CEU |
|  | rs1800629 | 6 | 31651010 | -2.031746679 | CEU |
|  | rs2066844 | 16 | 49303427 | -2.385525383 | CEU |
|  | rs2476 | 12 | 27077153 | -2.388969809 | CEU |
|  | rs2834213 | 21 | 33714780 | -2.065743351 | CEU |
|  | rs309132 | 2 | 136466743 | -2.319911461 | CEU |
|  | rs3091324 | 17 | 29625029 | -2.204450534 | CEU |
|  | rs76 | 7 | 11386632 | -2.035993689 | CEU |
|  | rs1799 | 2 | 152480979 | -2.428606785 | CEU |
|  | rs2011 | 3 | 38271821 | -2.047605189 | CEU |
|  | rs10515 | 19 | 9384724 | -2.014698826 | YRI |
|  | rs11436 | 1 | 31506413 | -3.010397064 | YRI |
|  | rs2107 | 8 | 53719888 | -2.026786713 | YRI |
|  | rs2243 | 6 | 117844083 | -2.169717407 | YRI |
|  | rs231775 | 2 | 204558220 | -2.293442893 | YRI |
|  | rs3212227 | 5 | 158675528 | -2.094541803 | YRI |
|  | rs37648 | 7 | 110577955 | -2.593752433 | YRI |
|  | rs64 | 7 | 11391070 | -2.179078865 | YRI |
|  | rs758 | 4 | 141982278 | -2.018514554 | YRI |
|  | rs7581 | 9 | 108858802 | -2.668770283 | YRI |
