# Supplementary material for: HGV&TB: a comprehensive online resource on human genes and genetic variants associated with tuberculosis
Source: Database (Oxford). 2014 Dec 13;2014:bau112. doi: 10.1093/database/bau112 (PMC5630898; doi:10.1093/database/bau112)
Supplement: Supplementary Data [file bau112_Supplementary_Data.zip › Supplementary_Table_4.docx]

**Supplementary Table 4: Counts of 307 associated variations in different genomic loci**

| **Genomic Loci** | **Count** |
| --- | --- |
| Exonic | 101 |
| Intronic | 78 |
| Intergenic | 38 |
| Upstream | 27 |
| Unknown | 15 |
| 3’ UTR | 11 |
| 5’ UTR | 11 |
| Haplotype | 10 |
| Downstream | 5 |
| ncRNA intronic | 5 |
| Splicing | 2 |
| Upstream; Downstream | 1 |
| ncRNA exonic | 1 |
| ncRNA 5’UTR | 1 |
| 5’ Gene Flanking | 1 |
