# Supplementary figures and images for: HGV&TB: a comprehensive online resource on human genes and genetic variants associated with tuberculosis
Source: Database (Oxford). 2014 Dec 13;2014:bau112. doi: 10.1093/database/bau112 (PMC5630898; doi:10.1093/database/bau112)

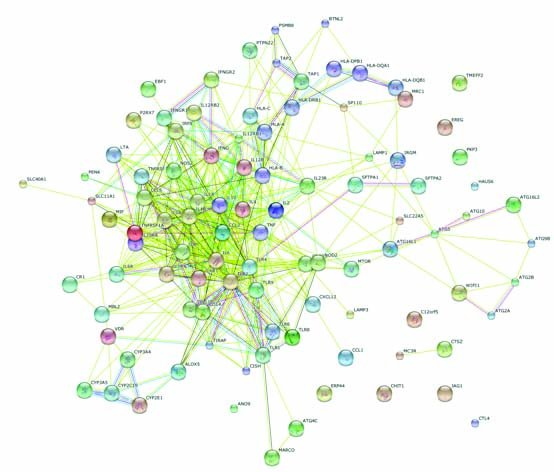

Supplement: Supplementary Data [file bau112_Supplementary_Data.zip › Supplementary_Figure_2.jpg]

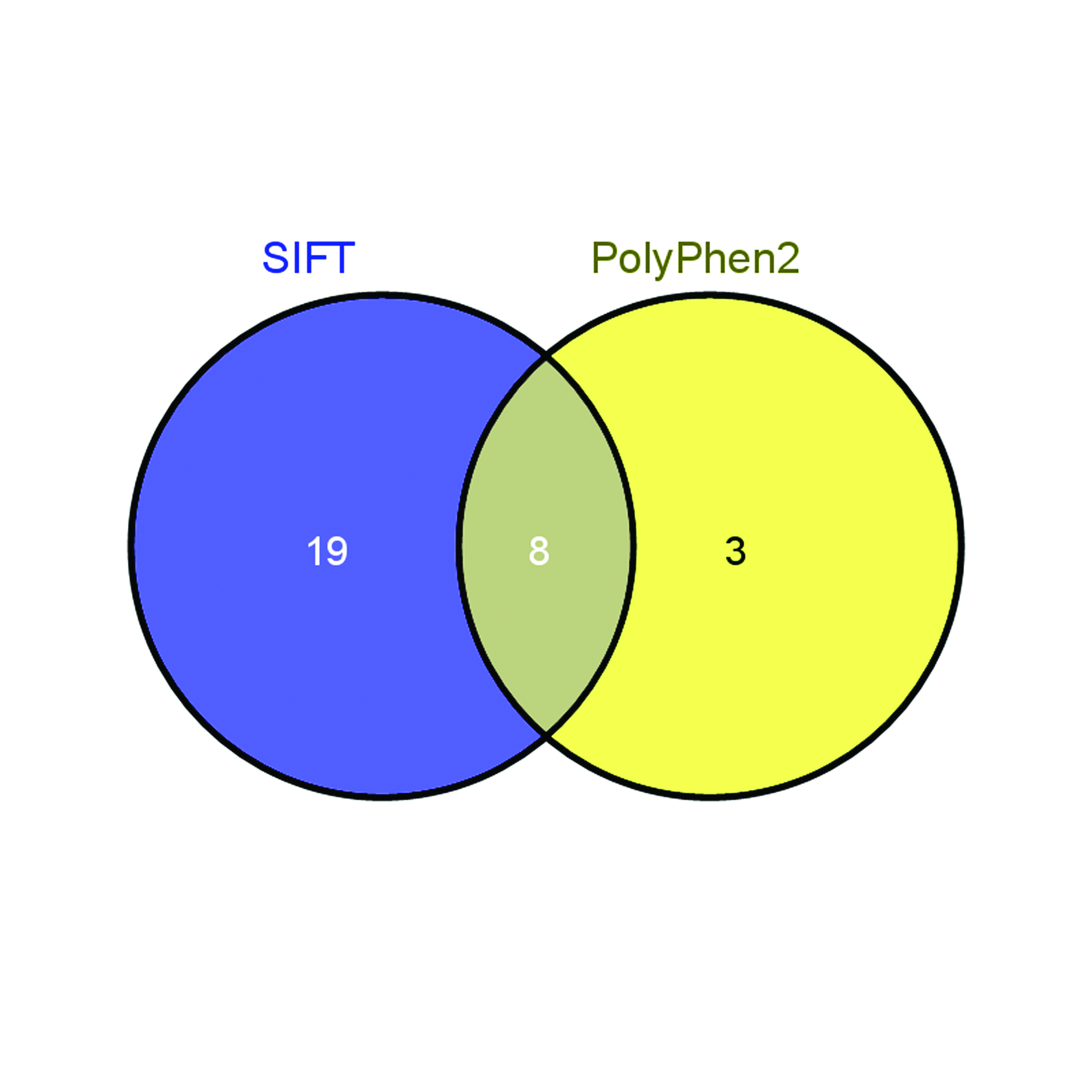

Supplement: Supplementary Data [file bau112_Supplementary_Data.zip › Supplementary_Figure_1.tif]

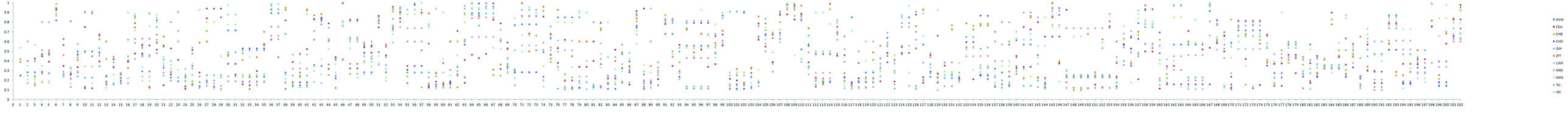

Supplement: Supplementary Data [file bau112_Supplementary_Data.zip › Supplementary_Figure_3.tif]
