# Supplementary material for: HGV&TB: a comprehensive online resource on human genes and genetic variants associated with tuberculosis
Source: Database (Oxford). 2014 Dec 13;2014:bau112. doi: 10.1093/database/bau112 (PMC5630898; doi:10.1093/database/bau112)
Supplement: Supplementary Data [file bau112_Supplementary_Data.zip › Supplementary_Table_1.doc]

**SUPPLEMENTARY TABLE 1: List of all the Tuberculosis vulnerable variant rsid’s associated with respective genes, having both individual and haplotype/diplotype/interaction association with Tuberculosis susceptibility uploaded in HGV&TB.**

| **S. No.** | **Gene** | **rsID** | **References** |
| --- | --- | --- | --- |
| 1. | AKT1 | rs3730358, rs1130233 |  |
| 2. | ALOX5 | rs2228065 |  |
| 3. | ANO9 | rs7111432 |  |
| 4. | ATG10 | rs1864183, rs3734114 |  |
| 5. | ATG16L1 | rs2241880 |  |
| 6. | ATG16L2 | rs11235604 |  |
| 7. | ATG2A | rs77228473, rs77833427 |  |
| 8. | ATG2B | rs9323945, rs74719094 |  |
| 9. | ATG4C | rs10493328, rs10493327 |  |
| 10. | ATG5 | rs2245214 |  |
| 11. | ATG9B | rs61733329 |  |
| 12. | BTNL2 | rs3763313, rs9268494, rs9268492, rs9405098, rs3763317, rs2076530 |  |
| 13. | CCL1 | rs10491110, rs3091324, rs2072069, rs159319, rs3138031, rs159291, rs159294, rs210837, rs159290 |  |
| 14. | CCL2 | rs1024611, rs4586, rs1799750, |  |
| 15. | CCL5 | rs2107538,rs2280788, rs2280789 |  |
| 16. | CD14 | _ |  |
| 17. | CD209 | rs4804803, rs735240 |  |
| 18. | CHIT1 | rs9943208, rs1065761 |  |
| 19. | CISH | _ |  |
| 20. | CR1 | _ |  |
| 21. | CTL4 | rs231775, rs3087243, rs11571319 |  |
| 22. | CTSZ | rs34069356, rs10369, rs9760, rs163790, rs163800, rs163801 |  |
| 23. | CXCL12 | rs2839693, rs1801157 |  |
| 24. | CYP2C19 | rs4986893, rs4244285 |  |
| 25. | CYP2E1 | rs2031920, rs6413432 |  |
| 26. | CYP3A4 | rs28371759 |  |
| 27. | CYP3A5 | rs776746 |  |
| 28. | EBF1 | rs10515787 |  |
| 29. | EREG | rs7675690 |  |
| 30. | ERP44 | rs1418267 |  |
| 31. | HAUS6 | rs10245298 |  |
| 32. | HLA-A | _ |  |
| 33. | HLA-B | _ |  |
| 34. | HLA-C | _ |  |
| 35. | HLA-DPB1 | _ |  |
| 36. | HLA-DQA1 | _ |  |
| 37. | HLA-DQB1 | rs9273665 |  |
| 38. | HLA-DRB1 | _ |  |
| 39. | IFNG | rs2430561, rs1861493, rs1861494 |  |
| 40. | IFNGR1 | rs2430561, rs2234711, rs1327474, rs7749390, rs4896243, rs1861493, rs1861494, rs41401746, rs2248814, rs3729508, rs3729718, rs2274894, rs2314809, rs944722, rs7215373 |  |
| 41. | IFNGR2 | rs2430561, rs2834213, rs1059293, rs1861493, rs1861494 |  |
| 42. | IL10 | rs1800896, rs1800872, rs1518111, rs1554286, rs1800870, rs1800871 |  |
| 43. | IL10RA | rs1800896, rs1800872, rs1518111, rs1554286, rs1800870, rs3135932, rs1800871 |  |
| 44. | IL12B | rs3212227, rs11574790, rs3212220, rs2853694 |  |
| 45. | IL12RB1 | rs11575934, rs375947, rs401502 |  |
| 46. | IL12RB2 | rs11810249 |  |
| 47. | IL18 | rs1946518 |  |
| 48. | IL1A | rs1800587 |  |
| 49. | IL1B | rs1143634 |  |
| 50. | IL1R1 | _ |  |
| 51. | IL1RN | rs4252019 |  |
| 52. | IL2 | _ |  |
| 53. | IL23R | rs11209026 |  |
| 54. | IL4 | rs2243250, rs2070874 |  |
| 55. | IL6 | rs1800795, rs13306436, rs2069824, rs36215814, rs1800797, rs1800796, rs56077270 |  |
| 56. | IL6R | rs1800795, rs13306436, rs2069824, rs36215814, rs1800797, rs1800796, rs56077270, rs1552481, rs2229238, rs3887104, rs4379670 |  |
| 57. | IL8 | _ |  |
| 58. | IRF8 | rs925994, rs11117415, rs10514611 |  |
| 59. | IRGM | rs10065172, rs4958842, rs4958843, rs4958846, rs72553867, rs4958847 |  |
| 60. | JAG1 | rs2273061 |  |
| 61. | LAMP1 | rs9577229 |  |
| 62. | LAMP3 | rs482912 |  |
| 63. | LTA | rs361525, rs1800629, rs1041981, rs7791836 , rs1399431, rs2229094 |  |
| 64. | MARCO | rs17009726, rs2278588, rs17795618, rs1371562, rs6761637, rs2011839 |  |
| 65. | MBL2 | rs1800451, rs5030737, rs1800450, rs1800405 |  |
| 66. | MC3R | rs3827103, rs6127698 |  |
| 67. | MIF | rs755622 |  |
| 68. | MRC1 | rs34039386, rs71497223, rs71497224, rs34284571, _ |  |
| 69. | MTOR | rs6701524, rs10492975 |  |
| 70. | NOD2 | rs2066842, rs2066844, rs5743278, rs7194886 |  |
| 71. | NOS2 | rs2274894, rs7215373, rs2255929, rs944722, rs2314809, rs3729718, rs2248814, rs3729508, rs1327474, rs5030729, rs2274894,rs5030729, rs7234985, rs57234985, rs9282799, rs8073782 |  |
| 72. | P2RX7 | rs1653624 , rs3751143, rs2393799 |  |
| 73. | PENK | rs6985962 |  |
| 74. | PKP3 | rs10902158, rs7105848 |  |
| 75. | PSMB8 | rs2071543, _ |  |
| 76. | PTPN22 | rs2476601, rs33996649 |  |
| 77. | SFTPA1 | rs1136450, rs72659390, rs1136451 |  |
| 78. | SFTPA2 | rs17886395, rs17886221, rs1965708 |  |
| 79. | SLC11A1 | rs17235409, rs3731865, rs2276631, rs3731863, rs17221959, rs34448891, rs17229009, rs17235416, rs1816702 |  |
| 80. | SLC22A5 | rs274559, rs274554, rs274553 |  |
| 81. | SLC40A1 | rs10188230, rs10188680, rs10202029, rs11568344, rs11568350, rs116496357, rs11884632, rs13008848, rs13012833, rs1439814, rs1439816, rs1568351, rs2304704, rs2352262, rs3792079, rs3811621, rs61525883, rs6706281, rs994226 |  |
| 82. | SP110 | rs2114592, rs3948464, rs9061, rs1135791, rs722555, rs11679983, rs7581442, rs7573954, rs13389060, rs11556887 |  |
| 83. | TAP1 | _ |  |
| 84. | TAP2 | rs1135216 |  |
| 85. | TIRAP | rs7932766, rs8177374, rs8177400, rs3804099,rs7932766, rs3802814, rs3802813 |  |
| 86. | TLR1 | rs4833095, rs76798247, rs5743810 |  |
| 87. | TLR2 | rs5743708, rs3804099, rs3804100, rs3731865, rs1816702, rs4696480, rs1898830, rs7932766 |  |
| 88. | TLR4 | rs4986790, rs5030729, rs2248814, rs1399431, rs7791836, rs2274894, rs7215373 |  |
| 89. | TLR6 | rs4833095, rs76798247, rs5743810 |  |
| 90. | TLR8 | rs3764879, rs3788935, rs3761624, rs3764880 |  |
| 91. | TLR9 | rs164637, rs352143, rs352139, rs5743836 |  |
| 92. | TMEFF2 | rs10497744, rs1020941 |  |
| 93. | TNF | rs361525, rs1800629, rs7791836 , rs1399431 |  |
| 94. | TNFRSF1A | rs361525, rs1800629, rs4149623, rs4149639, rs4149622, rs4149578, rs7791836 , rs1399431 |  |
| 95. | TNFRSF1B | rs361525, rs1800629, rs496888, rs1061624, rs7791836 , rs1399431 |  |
| 96. | TOLLIP | rs3750920, rs5743899 |  |
| 97. | VDR | rs7975232, rs2228570, rs1544410, rs731236, rs11568820, rs4516035 |  |
| 98. | WIPI1 | rs883541 |  |

**References:**

1.	Wang X Fau - Cao, Z., Cao Z Fau - Jiang, J., Jiang J Fau - Zhu, Y.*, et al.* (2010) AKT1 polymorphisms are associated with tuberculosis in the Chinese population. *Int J Immunogenet*, **37**, 97-101 LID - 110.1111/j.1744-1313X.2010.00897.x [doi].

2.	Herb F Fau - Thye, T., Thye T Fau - Niemann, S., Niemann S Fau - Browne, E.N.L.*, et al.* (2008) ALOX5 variants associated with susceptibility to human pulmonary tuberculosis. *Hum Mol Genet*, **17**, 1052-1060 LID - 1010.1093/hmg/ddm1378 [doi].

3.	Horne, D.J., Randhawa, A.K., Chau, T.T.*, et al.* (2012) Common polymorphisms in the PKP3-SIGIRR-TMEM16J gene region are associated with susceptibility to tuberculosis. *The Journal of infectious diseases*, **205**, 586-594.

4.	Songane, M., Kleinnijenhuis, J., Alisjahbana, B.*, et al.* (2012) Polymorphisms in autophagy genes and susceptibility to tuberculosis. *PloS one*, **7**, e41618.

5.	Lian Y Fau - Yue, J., Yue J Fau - Han, M., Han M Fau - Liu, J.*, et al.* (2010) Analysis of the association between BTNL2 polymorphism and tuberculosis in. *Infect Genet Evol*, **10**, 517-521 LID - 510.1016/j.meegid.2010.1002.1006 [doi].

6.	Thuong Nt Fau - Dunstan, S.J., Dunstan Sj Fau - Chau, T.T.H., Chau Tt Fau - Thorsson, V.*, et al.* (2008) Identification of tuberculosis susceptibility genes with human macrophage gene. *PLoS Pathog*, **4**, e1000229 LID - 1000210.1001371/journal.ppat.1000229 [doi].

7.	Ben-Selma W Fau - Ben-Abderrahmen, Y., Ben-Abderrahmen Y Fau - Boukadida, J., Boukadida J Fau - Harizi, H.*, et al.* (2012) IL-10R1 S138G loss-of-function polymorphism is associated with extrapulmonary. *Mol Biol Rep*, **39**, 51-56 LID - 10.1007/s11033-11011-10709-11032 [doi].

8.	Buijtels Pc Fau - van de Sande, W.W.J., van de Sande Ww Fau - Parkinson, S., Parkinson S Fau - Petit, P.L.C.*, et al.* (2008) Polymorphism in CC-chemokine ligand 2 associated with tuberculosis in Zambia. *Int J Tuberc Lung Dis*, **12**, 1485-1488.

9.	Feng Wx Fau - Mokrousov, I., Mokrousov I Fau - Wang, B.-B., Wang Bb Fau - Nelson, H.*, et al.* (2011) Tag SNP polymorphism of CCL2 and its role in clinical tuberculosis in Han Chinese. *PloS one*, **6**, e14652 LID - 14610.11371/journal.pone.0014652 [doi].

10.	Flores-Villanueva Po Fau - Ruiz-Morales, J.A., Ruiz-Morales Ja Fau - Song, C.-H., Song Ch Fau - Flores, L.M.*, et al.* (2005) A functional promoter polymorphism in monocyte chemoattractant protein-1 is. *J Exp Med*, **202**, 1649-1658.

11.	Ganachari M Fau - Ruiz-Morales, J.A., Ruiz-Morales Ja Fau - Gomez de la Torre Pretell, J.C., Gomez de la Torre Pretell Jc Fau - Dinh, J.*, et al.* (2010) Joint effect of MCP-1 genotype GG and MMP-1 genotype 2G/2G increases the. *PloS one*, **5**, e8881 LID - 8810.1371/journal.pone.0008881 [doi].

12.	Yang L Fau - Shi, G.L., Shi Gl Fau - Song, C.X., Song Cx Fau - Xu, S.F.*, et al.* (2010) Relationship between genetic polymorphism of MCP-1 and non-small-cell lung cancer. *Genet Mol Res*, **9**, 765-771 LID - 710.4238/vol4239-4232gmr4740 [doi].

13.	Ben-Selma W Fau - Harizi, H., Harizi H Fau - Bougmiza, I., Bougmiza I Fau - Ben Kahla, I.*, et al.* (2011) Polymorphisms in the RANTES gene increase susceptibility to active tuberculosis. *DNA Cell Biol*, **30**, 789-800 LID - 710.1089/dna.2010.1200 [doi].

14.	Chu Sf Fau - Tam, C.M., Tam Cm Fau - Wong, H.S., Wong Hs Fau - Kam, K.M.*, et al.* (2007) Association between RANTES functional polymorphisms and tuberculosis in Hong Kong. *Genes Immun*, **8**, 475-479.

15.	Sanchez-Castanon M Fau - Baquero, I.C., Baquero Ic Fau - Sanchez-Velasco, P., Sanchez-Velasco P Fau - Farinas, M.C.*, et al.* (2009) Polymorphisms in CCL5 promoter are associated with pulmonary tuberculosis in. *Int J Tuberc Lung Dis*, **13**, 480-485.

16.	Sharma Pr Fau - Singh, S., Singh S Fau - Jena, M., Jena M Fau - Mishra, G.*, et al.* (2011) Coding and non-coding polymorphisms in VDR gene and susceptibility to pulmonary. *Infect Genet Evol*, **11**, 1456-1461 LID - 1410.1016/j.meegid.2011.1405.1019 [doi].

17.	Kang, Y.A., Lee, H.W., Kim, Y.W.*, et al.* (2009) Association between the -159C/T CD14 gene polymorphism and tuberculosis in a Korean population. *FEMS immunology and medical microbiology*, **57**, 229-235.

18.	Rosas-Taraco, A.G., Revol, A., Salinas-Carmona, M.C.*, et al.* (2007) CD14 C(-159)T polymorphism is a risk factor for development of pulmonary tuberculosis. *The Journal of infectious diseases*, **196**, 1698-1706.

19.	Kobayashi K Fau - Yuliwulandari, R., Yuliwulandari R Fau - Yanai, H., Yanai H Fau - Lien, L.T.*, et al.* (2011) Association of CD209 polymorphisms with tuberculosis in an Indonesian population. *Hum Immunol*, **72**, 741-745 LID - 710.1016/j.humimm.2011.1004.1004 [doi].

20.	Selvaraj P Fau - Alagarasu, K., Alagarasu K Fau - Swaminathan, S., Swaminathan S Fau - Harishankar, M.*, et al.* (2009) CD209 gene polymorphisms in South Indian HIV and HIV-TB patients. *Infect Genet Evol*, **9**, 256-262 LID - 210.1016/j.meegid.2008.1012.1003 [doi].

21.	Lee P Fau - Waalen, J., Waalen J Fau - Crain, K., Crain K Fau - Smargon, A.*, et al.* (2007) Human chitotriosidase polymorphisms G354R and A442V associated with reduced. *Blood Cells Mol Dis*, **39**, 353-360.

22.	Khor, C.C., Vannberg, F.O., Chapman, S.J.*, et al.* (2010) CISH and susceptibility to infectious diseases. *The New England journal of medicine*, **362**, 2092-2101.

23.	Fitness J Fau - Floyd, S., Floyd S Fau - Warndorff, D.K., Warndorff Dk Fau - Sichali, L.*, et al.* (2004) Large-scale candidate gene study of tuberculosis susceptibility in the Karonga. *Am J Trop Med Hyg*, **71**, 341-349.

24.	Tso Hw Fau - Ip, W.K., Ip Wk Fau - Chong, W.P., Chong Wp Fau - Tam, C.M.*, et al.* (2005) Association of interferon gamma and interleukin 10 genes with tuberculosis in. *Genes Immun*, **6**, 358-363.

25.	Adams La Fau - Moller, M., Moller M Fau - Nebel, A., Nebel A Fau - Schreiber, S.*, et al.* (2011) Polymorphisms in MC3R promoter and CTSZ 3'UTR are associated with tuberculosis

A method and server for predicting damaging missense mutations. *Eur J Hum Genet*, **19**, 676-681 LID - 610.1038/ejhg.2011.1031 [doi].

26.	Baker Ar Fau - Zalwango, S., Zalwango S Fau - Malone, L.L., Malone Ll Fau - Igo, R.P., Jr.*, et al.* (2011) Genetic susceptibility to tuberculosis associated with cathepsin Z haplotype in a. *Hum Immunol*, **72**, 426-430 LID - 410.1016/j.humimm.2011.1002.1016 [doi].

27.	Cooke Gs Fau - Campbell, S.J., Campbell Sj Fau - Bennett, S., Bennett S Fau - Lienhardt, C.*, et al.* (2008) Mapping of a novel susceptibility locus suggests a role for MC3R and CTSZ in. *Am J Respir Crit Care Med*, **178**, 203-207 LID - 210.1164/rccm.200710-201554OC [doi].

28.	Selvaraj P Fau - Alagarasu, K., Alagarasu K Fau - Harishankar, M., Harishankar M Fau - Vidyarani, M.*, et al.* (2008) Regulatory region polymorphisms of vitamin D receptor gene in pulmonary. *Int J Immunogenet*, **35**, 251-254 LID - 210.1111/j.1744-1313X.2008.00764.x [doi].

29.	Selvaraj P Fau - Alagarasu, K., Alagarasu K Fau - Singh, B., Singh, B. (2012) Stromal cell-derived factor-1 (SDF-1/CXCL12) gene polymorphisms in pulmonary. *Int J Immunogenet*, **39**, 26-31 LID - 10.1111/j.1744-1313X.2011.01059.x [doi].

30.	Png, E., Alisjahbana, B., Sahiratmadja, E.*, et al.* (2012) A genome wide association study of pulmonary tuberculosis susceptibility in Indonesians. *BMC medical genetics*, **13**, 5.

31.	Figueiredo, J.F., Rodrigues Mde, L., Deghaide, N.H.*, et al.* (2008) HLA profile in patients with AIDS and tuberculosis. *The Brazilian journal of infectious diseases : an official publication of the Brazilian Society of Infectious Diseases*, **12**, 278-280.

32.	Raghavan, S., Selvaraj, P., Swaminathan, S.*, et al.* (2009) Haplotype analysis of HLA-A, -B antigens and -DRB1 alleles in south Indian HIV-1-infected patients with and without pulmonary tuberculosis. *Int J Immunogenet*, **36**, 129-133.

33.	Louie, L.G., Hartogensis, W.E., Jackman, R.P.*, et al.* (2004) Mycobacterium tuberculosis/HIV-1 coinfection and disease: role of human leukocyte antigen variation. *The Journal of infectious diseases*, **189**, 1084-1090.

34.	Teran-Escandon, D., Teran-Ortiz, L., Camarena-Olvera, A.*, et al.* (1999) Human leukocyte antigen-associated susceptibility to pulmonary tuberculosis: molecular analysis of class II alleles by DNA amplification and oligonucleotide hybridization in Mexican patients. *Chest*, **115**, 428-433.

35.	Delgado, J.C., Baena, A., Thim, S.*, et al.* (2006) Aspartic acid homozygosity at codon 57 of HLA-DQ beta is associated with susceptibility to pulmonary tuberculosis in Cambodia. *Journal of immunology*, **176**, 1090-1097.

36.	Dubaniewicz, A., Moszkowska, G., Szczerkowska, Z. (2005) Frequency of DRB1-DQB1 two-locus haplotypes in tuberculosis: preliminary report. *Tuberculosis*, **85**, 259-267.

37.	Goldfeld, A.E., Delgado, J.C., Thim, S.*, et al.* (1998) Association of an HLA-DQ allele with clinical tuberculosis. *JAMA : the journal of the American Medical Association*, **279**, 226-228.

38.	Kim, H.S., Park, M.H., Song, E.Y.*, et al.* (2005) Association of HLA-DR and HLA-DQ genes with susceptibility to pulmonary tuberculosis in Koreans: preliminary evidence of associations with drug resistance, disease severity, and disease recurrence. *Hum Immunol*, **66**, 1074-1081.

39.	Ravikumar, M., Dheenadhayalan, V., Rajaram, K.*, et al.* (1999) Associations of HLA-DRB1, DQB1 and DPB1 alleles with pulmonary tuberculosis in south India. *Tubercle and lung disease : the official journal of the International Union against Tuberculosis and Lung Disease*, **79**, 309-317.

40.	Amirzargar Aa Fau - Rezaei, N., Rezaei N Fau - Jabbari, H., Jabbari H Fau - Danesh, A.-A.*, et al.* (2006) Cytokine single nucleotide polymorphisms in Iranian patients with pulmonary. *Eur Cytokine Netw*, **17**, 84-89.

41.	Duarte, R., Carvalho, C., Pereira, C.*, et al.* (2011) HLA class II alleles as markers of tuberculosis susceptibility and resistance. *Revista portuguesa de pneumologia*, **17**, 15-19.

42.	Abhimanyu, Bose, M., Jha, P.*, et al.* (2012) Footprints of genetic susceptibility to pulmonary tuberculosis: cytokine gene variants in north Indians. *The Indian journal of medical research*, **135**, 763-770.

43.	Amim, L.H., Pacheco, A.G., Fonseca-Costa, J.*, et al.* (2008) Role of IFN-gamma +874 T/A single nucleotide polymorphism in the tuberculosis outcome among Brazilians subjects. *Mol Biol Rep*, **35**, 563-566.

44.	Ansari, A., Talat, N., Jamil, B.*, et al.* (2009) Cytokine gene polymorphisms across tuberculosis clinical spectrum in Pakistani patients. *PloS one*, **4**, e4778.

45.	Henao, M.I., Montes, C., Paris, S.C.*, et al.* (2006) Cytokine gene polymorphisms in Colombian patients with different clinical presentations of tuberculosis. *Tuberculosis*, **86**, 11-19.

46.	Lopez-Maderuelo, D., Arnalich, F., Serantes, R.*, et al.* (2003) Interferon-gamma and interleukin-10 gene polymorphisms in pulmonary tuberculosis. *Am J Respir Crit Care Med*, **167**, 970-975.

47.	Rossouw, M., Nel, H.J., Cooke, G.S.*, et al.* (2003) Association between tuberculosis and a polymorphic NFkappaB binding site in the interferon gamma gene. *Lancet*, **361**, 1871-1872.

48.	Sallakci, N., Coskun, M., Berber, Z.*, et al.* (2007) Interferon-gamma gene+874T-A polymorphism is associated with tuberculosis and gamma interferon response. *Tuberculosis*, **87**, 225-230.

49.	Tso, H.W., Ip, W.K., Chong, W.P.*, et al.* (2005) Association of interferon gamma and interleukin 10 genes with tuberculosis in Hong Kong Chinese. *Genes Immun*, **6**, 358-363.

50.	Stein, C.M., Zalwango, S., Chiunda, A.B.*, et al.* (2007) Linkage and association analysis of candidate genes for TB and TNFalpha cytokine expression: evidence for association with IFNGR1, IL-10, and TNF receptor 1 genes. *Human genetics*, **121**, 663-673.

51.	He, J., Wang, J., Lei, D.*, et al.* (2010) Analysis of functional SNP in ifng/ifngr1 in Chinese Han population with tuberculosis. *Scandinavian journal of immunology*, **71**, 452-458.

52.	Velez, D.R., Hulme, W.F., Myers, J.L.*, et al.* (2009) NOS2A, TLR4, and IFNGR1 interactions influence pulmonary tuberculosis susceptibility in African-Americans. *Human genetics*, **126**, 643-653.

53.	Hijikata, M., Shojima, J., Matsushita, I.*, et al.* (2012) Association of IFNGR2 gene polymorphisms with pulmonary tuberculosis among the Vietnamese. *Human genetics*, **131**, 675-682.

54.	Ates O Fau - Musellim, B., Musellim B Fau - Ongen, G., Ongen G Fau - Topal-Sarikaya, A.*, et al.* (2008) Interleukin-10 and tumor necrosis factor-alpha gene polymorphisms in. *J Clin Immunol*, **28**, 232-236.

55.	Ben-Selma W Fau - Harizi, H., Harizi H Fau - Boukadida, J., Boukadida, J. (2011) Association of TNF-alpha and IL-10 polymorphisms with tuberculosis in Tunisian. *Microbes Infect*, **13**, 837-843 LID - 810.1016/j.micinf.2011.1004.1009 [doi].

56.	Guwatudde, D., Zalwango, S., Kamya, M.R.*, et al.* (2003) Burden of tuberculosis in Kampala, Uganda. *Bulletin of the World Health Organization*, **81**, 799-805.

57.	Oh Jh Fau - Yang, C.-S., Yang Cs Fau - Noh, Y.-K., Noh Yk Fau - Kweon, Y.-M.*, et al.* (2007) Polymorphisms of interleukin-10 and tumour necrosis factor-alpha genes are. *Respirology*, **12**, 594-598.

58.	Oral Hb Fau - Budak, F., Budak F Fau - Uzaslan, E.K., Uzaslan Ek Fau - Basturk, B.*, et al.* (2006) Interleukin-10 (IL-10) gene polymorphism as a potential host susceptibility. *Cytokine*, **35**, 143-147.

59.	Stein Cm Fau - Zalwango, S., Zalwango S Fau - Chiunda, A.B., Chiunda Ab Fau - Millard, C.*, et al.* (2007) Linkage and association analysis of candidate genes for TB and TNFalpha cytokine. *Human genetics*, **121**, 663-673.

60.	Taype, C.A., Shamsuzzaman, S., Accinelli, R.A.*, et al.* (2010) Genetic susceptibility to different clinical forms of tuberculosis in the Peruvian population. *Infect Genet Evol*, **10**, 495-504.

61.	Morris Ga Fau - Edwards, D.R.V., Edwards Dr Fau - Hill, P.C., Hill Pc Fau - Wejse, C.*, et al.* (2011) Interleukin 12B (IL12B) genetic variation and pulmonary tuberculosis: a study of. *PloS one*, **6**, e16656 LID - 16610.11371/journal.pone.0016656 [doi].

62.	Akahoshi M Fau - Nakashima, H., Nakashima H Fau - Miyake, K., Miyake K Fau - Inoue, Y.*, et al.* (2003) Influence of interleukin-12 receptor beta1 polymorphisms on tuberculosis. *Human genetics*, **112**, 237-243.

63.	Kusuhara, K., Yamamoto, K., Okada, K.*, et al.* (2007) Association of IL12RB1 polymorphisms with susceptibility to and severity of tuberculosis in Japanese: a gene-based association analysis of 21 candidate genes. *Int J Immunogenet*, **34**, 35-44.

64.	Remus, N., El Baghdadi, J., Fieschi, C.*, et al.* (2004) Association of IL12RB1 polymorphisms with pulmonary tuberculosis in adults in Morocco. *The Journal of infectious diseases*, **190**, 580-587.

65.	Verma, V.K., Taneja, V., Jaiswal, A.*, et al.* (2012) Prevalence, distribution and functional significance of the -237C to T polymorphism in the IL-12Rbeta2 promoter in Indian tuberculosis patients. *PloS one*, **7**, e34355.

66.	Taheri, M., Hashemi-Shahri, S.M., Hamzehnejadi, M.*, et al.* (2012) Lack of association between interleukin-18 -607 C/A gene polymorphism and pulmonary tuberculosis in Zahedan, Southeast Iran. *Prague medical report*, **113**, 16-22.

67.	Naslednikova Io Fau - Urazova, O.I., Urazova Oi Fau - Voronkova, O.V., Voronkova Ov Fau - Strelis, A.K.*, et al.* (2009) Allelic polymorphism of cytokine genes during pulmonary tuberculosis. *Bull Exp Biol Med*, **148**, 175-180.

68.	Vidyarani, M., Selvaraj, P., Prabhu Anand, S.*, et al.* (2006) Interferon gamma (IFNgamma) & interleukin-4 (IL-4) gene variants & cytokine levels in pulmonary tuberculosis. *The Indian journal of medical research*, **124**, 403-410.

69.	Zhang, G., Zhou, B., Wang, W.*, et al.* (2012) A functional single-nucleotide polymorphism in the promoter of the gene encoding interleukin 6 is associated with susceptibility to tuberculosis. *The Journal of infectious diseases*, **205**, 1697-1704.

70.	Ma, X., Reich, R.A., Wright, J.A.*, et al.* (2003) Association between interleukin-8 gene alleles and human susceptibility to tuberculosis disease. *The Journal of infectious diseases*, **188**, 349-355.

71.	Ding, S., Jiang, T., He, J.*, et al.* (2012) Tagging single nucleotide polymorphisms in the IRF1 and IRF8 genes and tuberculosis susceptibility. *PloS one*, **7**, e42104.

72.	Bahari, G., Hashemi, M., Taheri, M.*, et al.* (2012) Association of IRGM polymorphisms and susceptibility to pulmonary tuberculosis in Zahedan, Southeast Iran. *TheScientificWorldJournal*, **2012**, 950801.

73.	Che, N., Li, S., Gao, T.*, et al.* (2010) Identification of a novel IRGM promoter single nucleotide polymorphism associated with tuberculosis. *Clinica chimica acta; international journal of clinical chemistry*, **411**, 1645-1649.

74.	King, K.Y., Lew, J.D., Ha, N.P.*, et al.* (2011) Polymorphic allele of human IRGM1 is associated with susceptibility to tuberculosis in African Americans. *PloS one*, **6**, e16317.

75.	Taype Ca Fau - Shamsuzzaman, S., Shamsuzzaman S Fau - Accinelli, R.A., Accinelli Ra Fau - Espinoza, J.R.*, et al.* (2010) Genetic susceptibility to different clinical forms of tuberculosis in the. *Infect Genet Evol*, **10**, 495-504 LID - 410.1016/j.meegid.2010.1002.1011 [doi].

76.	Ma Mj Fau - Wang, H.-B., Wang Hb Fau - Li, H., Li H Fau - Yang, J.-H.*, et al.* (2011) Genetic variants in MARCO are associated with the susceptibility to pulmonary. *PloS one*, **6**, e24069 LID - 24010.21371/journal.pone.0024069 [doi].

77.	Alagarasu, K., Selvaraj, P., Swaminathan, S.*, et al.* (2007) Mannose binding lectin gene variants and susceptibility to tuberculosis in HIV-1 infected patients of South India. *Tuberculosis*, **87**, 535-543.

78.	Capparelli, R., Iannaccone, M., Palumbo, D.*, et al.* (2009) Role played by human mannose-binding lectin polymorphisms in pulmonary tuberculosis. *The Journal of infectious diseases*, **199**, 666-672.

79.	Feng, F.M., Guo, M., Liu, Q.*, et al.* (2006) [Study on mannose-binding protein gene polymorphisms and susceptibility to pulmonary tuberculosis]. *Zhonghua liu xing bing xue za zhi = Zhonghua liuxingbingxue zazhi*, **27**, 1082-1085.

80.	Liu W Fau - Zhang, C.-y., Zhang Cy Fau - Tian, L., Tian L Fau - Li, C.-z.*, et al.* (2003) [A case-control study on natural-resistance-associated macrophage protein 1 gene. *Zhonghua Yu Fang Yi Xue Za Zhi*, **37**, 408-411.

81.	Selvaraj, P., Narayanan, P.R., Reetha, A.M. (1999) Association of functional mutant homozygotes of the mannose binding protein gene with susceptibility to pulmonary tuberculosis in India. *Tubercle and lung disease : the official journal of the International Union against Tuberculosis and Lung Disease*, **79**, 221-227.

82.	Adams La Fau - Moller, M., Moller M Fau - Nebel, A., Nebel A Fau - Schreiber, S.*, et al.* (2011) Polymorphisms in MC3R promoter and CTSZ 3'UTR are associated with tuberculosis. *Eur J Hum Genet*, **19**, 676-681 LID - 610.1038/ejhg.2011.1031 [doi].

83.	Gomez, L.M., Sanchez, E., Ruiz-Narvaez, E.A.*, et al.* (2007) Macrophage migration inhibitory factor gene influences the risk of developing tuberculosis in northwestern Colombian population. *Tissue antigens*, **70**, 28-33.

84.	Sadki, K., Lamsyah, H., Rueda, B.*, et al.* (2010) Analysis of MIF, FCGR2A and FCGR3A gene polymorphisms with susceptibility to pulmonary tuberculosis in Moroccan population. *Journal of genetics and genomics = Yi chuan xue bao*, **37**, 257-264.

85.	Zhang, X., Jiang, F., Wei, L.*, et al.* (2012) Polymorphic allele of human MRC1 confer protection against tuberculosis in a Chinese population. *International journal of biological sciences*, **8**, 375-382.

86.	Austin, C.M., Ma, X., Graviss, E.A. (2008) Common nonsynonymous polymorphisms in the NOD2 gene are associated with resistance or susceptibility to tuberculosis disease in African Americans. *The Journal of infectious diseases*, **197**, 1713-1716.

87.	Pan, H., Dai, Y., Tang, S.*, et al.* (2012) Polymorphisms of NOD2 and the risk of tuberculosis: a validation study in the Chinese population. *Int J Immunogenet*, **39**, 233-240.

88.	Moller, M., Nebel, A., Valentonyte, R.*, et al.* (2009) Investigation of chromosome 17 candidate genes in susceptibility to TB in a South African population. *Tuberculosis*, **89**, 189-194.

89.	Ben-Selma W Fau - Ben-Kahla, I., Ben-Kahla I Fau - Boukadida, J., Boukadida J Fau - Harizi, H.*, et al.* (2011) Contribution of the P2X7 1513A/C loss-of-function polymorphism to extrapulmonary. *FEMS immunology and medical microbiology*, **63**, 65-72 LID - 10.1111/j.1574-1695X.2011.00824.x [doi].

90.	Fernando, S.L., Saunders, B.M., Sluyter, R.*, et al.* (2007) A polymorphism in the P2X7 gene increases susceptibility to extrapulmonary tuberculosis. *Am J Respir Crit Care Med*, **175**, 360-366.

91.	Nino-Moreno, P., Portales-Perez, D., Hernandez-Castro, B.*, et al.* (2007) P2X7 and NRAMP1/SLC11 A1 gene polymorphisms in Mexican mestizo patients with pulmonary tuberculosis. *Clinical and experimental immunology*, **148**, 469-477.

92.	Sambasivan V Fau - Murthy, K.J.R., Murthy Kj Fau - Reddy, R., Reddy R Fau - Vijayalakshimi, V.*, et al.* (2010) P2X7 gene polymorphisms and risk assessment for pulmonary tuberculosis in Asian. *Dis Markers*, **28**, 43-48 LID - 10.3233/DMA-2010-0682 [doi].

93.	Sharma, S., Kumar, V., Khosla, R.*, et al.* (2010) Association of P2X7 receptor +1513 (A-->C) polymorphism with tuberculosis in a Punjabi population. *Int J Tuberc Lung Dis*, **14**, 1159-1163.

94.	Singla N Fau - Gupta, D., Gupta D Fau - Joshi, A., Joshi A Fau - Batra, N.*, et al.* (2012) Genetic polymorphisms in the P2X7 gene and its association with susceptibility to. *Int J Tuberc Lung Dis*, **16**, 224-229 LID - 210.5588/ijtld.5511.0076 [doi].

95.	Wang, D., Zhou, Y., Ji, L.*, et al.* (2012) Association of LMP/TAP gene polymorphisms with tuberculosis susceptibility in Li population in China. *PloS one*, **7**, e33051.

96.	Gomez Lm Fau - Anaya, J.-M., Anaya Jm Fau - Martin, J., Martin, J. (2005) Genetic influence of PTPN22 R620W polymorphism in tuberculosis. *Hum Immunol*, **66**, 1242-1247.

97.	Kouhpayeh, H.R., Hashemi, M., Hashemi, S.A.*, et al.* (2012) R620W functional polymorphism of protein tyrosine phosphatase non-receptor type 22 is not associated with pulmonary tuberculosis in Zahedan, southeast Iran. *Genet Mol Res*, **11**, 1075-1081.

98.	Lamsyah H Fau - Rueda, B., Rueda B Fau - Baassi, L., Baassi L Fau - Elaouad, R.*, et al.* (2009) Association of PTPN22 gene functional variants with development of pulmonary. *Tissue antigens*, **74**, 228-232 LID - 210.1111/j.1399-0039.2009.01304.x [doi].

99.	Floros J Fau - Lin, H.M., Lin Hm Fau - Garcia, A., Garcia A Fau - Salazar, M.A.*, et al.* (2000) Surfactant protein genetic marker alleles identify a subgroup of tuberculosis in. *The Journal of infectious diseases*, **182**, 1473-1478.

100.	Madan T Fau - Saxena, S., Saxena S Fau - Murthy, K.J.R., Murthy Kj Fau - Muralidhar, K.*, et al.* (2002) Association of polymorphisms in the collagen region of human SP-A1 and SP-A2. *Clin Chem Lab Med*, **40**, 1002-1008.

101.	Malik S Fau - Abel, L., Abel L Fau - Tooker, H., Tooker H Fau - Poon, A.*, et al.* (2005) Alleles of the NRAMP1 gene are risk factors for pediatric tuberculosis disease. *Proc Natl Acad Sci U S A*, **102**, 12183-12188.

102.	Bellamy, R., Ruwende, C., Corrah, T.*, et al.* (1998) Variations in the NRAMP1 gene and susceptibility to tuberculosis in West Africans. *The New England journal of medicine*, **338**, 640-644.

103.	Jin, J., Sun, L., Jiao, W.W.*, et al.* (2009) [Association of NRAMP1 gene polymorphisms with the susceptibility to tuberculosis in ethnic Han Chinese children]. *Zhongguo dang dai er ke za zhi = Chinese journal of contemporary pediatrics*, **11**, 283-287.

104.	Liu, W., Zhang, C.Y., Tian, L.*, et al.* (2003) [A case-control study on natural-resistance-associated macrophage protein 1 gene polymorphisms and susceptibility to pulmonary tuberculosis]. *Zhonghua Yu Fang Yi Xue Za Zhi*, **37**, 408-411.

105.	Liu W Fau - Cao, W.C., Cao Wc Fau - Zhang, C.Y., Zhang Cy Fau - Tian, L.*, et al.* (2004) VDR and NRAMP1 gene polymorphisms in susceptibility to pulmonary tuberculosis. *Int J Tuberc Lung Dis*, **8**, 428-434.

106.	Soborg, C., Andersen, A.B., Range, N.*, et al.* (2007) Influence of candidate susceptibility genes on tuberculosis in a high endemic region. *Molecular immunology*, **44**, 2213-2220.

107.	Suzuki T Fau - Kameda, M., Kameda M Fau - Ando, M., Ando M Fau - Miyazoe, H.*, et al.* (2009) Discovery of novel diarylketoxime derivatives as selective and orally active. *Bioorg Med Chem Lett*, **19**, 5339-5345 LID - 5310.1016/j.bmcl.2009.5307.5132 [doi].

108.	Taype Ca Fau - Castro, J.C., Castro Jc Fau - Accinelli, R.A., Accinelli Ra Fau - Herrera-Velit, P.*, et al.* (2006) Association between SLC11A1 polymorphisms and susceptibility to different. *Infect Genet Evol*, **6**, 361-367.

109.	Velez Dr Fau - Hulme, W.F., Hulme Wf Fau - Myers, J.L., Myers Jl Fau - Stryjewski, M.E.*, et al.* (2009) Association of SLC11A1 with tuberculosis and interactions with NOS2A and TLR2 in. *Int J Tuberc Lung Dis*, **13**, 1068-1076.

110.	Zhang W Fau - Shao, L., Shao L Fau - Weng, X., Weng X Fau - Hu, Z.*, et al.* (2005) Variants of the natural resistance-associated macrophage protein 1 gene (NRAMP1). *Clin Infect Dis*, **40**, 1232-1236.

111.	Leung Kh Fau - Yip, S.P., Yip Sp Fau - Wong, W.S., Wong Ws Fau - Yiu, L.S.*, et al.* (2007) Sex- and age-dependent association of SLC11A1 polymorphisms with tuberculosis in. *BMC Infect Dis*, **7**, 19.

112.	McDermid, J.M., Hennig, B.J., van der Sande, M.*, et al.* (2013) Host iron redistribution as a risk factor for incident tuberculosis in HIV infection: an 11-year retrospective cohort study. *BMC Infect Dis*, **13**, 48.

113.	Ryu S Fau - Park, Y.K., Park Yk Fau - Bai, G.H., Bai Gh Fau - Kim, S.J.*, et al.* (2000) 3'UTR polymorphisms in the NRAMP1 gene are associated with susceptibility to. *Int J Tuberc Lung Dis*, **4**, 577-580.

114.	Velez, D.R., Hulme, W.F., Myers, J.L.*, et al.* (2009) Association of SLC11A1 with tuberculosis and interactions with NOS2A and TLR2 in African-Americans and Caucasians. *Int J Tuberc Lung Dis*, **13**, 1068-1076.

115.	Ridruechai C Fau - Mahasirimongkol, S., Mahasirimongkol S Fau - Phromjai, J., Phromjai J Fau - Yanai, H.*, et al.* (2010) Association analysis of susceptibility candidate region on chromosome 5q31 for. *Genes Immun*, **11**, 416-422 LID - 410.1038/gene.2010.1026 [doi].

116.	Baker, M.A., Wilson, D., Wallengren, K.*, et al.* (2012) Polymorphisms in the gene that encodes the iron transport protein ferroportin 1 influence susceptibility to tuberculosis. *The Journal of infectious diseases*, **205**, 1043-1047.

117.	Cong J Fau - Li, G., Li G Fau - Zhou, D., Zhou D Fau - Tao, Y.*, et al.* (2010) [Study on relation between Sp110 gene polymorphism and tuberculosis genetic. *Wei Sheng Yan Jiu*, **39**, 540-544.

118.	Liang L Fau - Zhao, Y.-l., Zhao Yl Fau - Yue, J., Yue J Fau - Liu, J.-f.*, et al.* (2011) Association of SP110 gene polymorphisms with susceptibility to tuberculosis in a. *Infect Genet Evol*, **11**, 934-939 LID - 910.1016/j.meegid.2011.1002.1017 [doi].

119.	Tosh K Fau - Campbell, S.J., Campbell Sj Fau - Fielding, K., Fielding K Fau - Sillah, J.*, et al.* (2006) Variants in the SP110 gene are associated with genetic susceptibility to. *Proc Natl Acad Sci U S A*, **103**, 10364-10368.

120.	Sunder, S.R., Hanumanth, S.R., Gaddam, S.*, et al.* (2011) Association of TAP 1 and 2 gene polymorphisms with human immunodeficiency virus-tuberculosis co-infection. *Hum Immunol*, **72**, 908-911.

121.	Dissanayeke, S.R., Levin, S., Pienaar, S.*, et al.* (2009) Polymorphic variation in TIRAP is not associated with susceptibility to childhood TB but may determine susceptibility to TBM in some ethnic groups. *PloS one*, **4**, e6698.

122.	Hawn, T.R., Dunstan, S.J., Thwaites, G.E.*, et al.* (2006) A polymorphism in Toll-interleukin 1 receptor domain containing adaptor protein is associated with susceptibility to meningeal tuberculosis. *The Journal of infectious diseases*, **194**, 1127-1134.

123.	Selvaraj, P., Harishankar, M., Singh, B.*, et al.* (2010) Toll-like receptor and TIRAP gene polymorphisms in pulmonary tuberculosis patients of South India. *Tuberculosis*, **90**, 306-310.

124.	Thuong, N.T., Hawn, T.R., Thwaites, G.E.*, et al.* (2007) A polymorphism in human TLR2 is associated with increased susceptibility to tuberculous meningitis. *Genes Immun*, **8**, 422-428.

125.	Zhang, Y.X., Xue, Y., Liu, J.Y.*, et al.* (2011) Association of TIRAP (MAL) gene polymorhisms with susceptibility to tuberculosis in a Chinese population. *Genet Mol Res*, **10**, 7-15.

126.	Ma, X., Liu, Y., Gowen, B.B.*, et al.* (2007) Full-exon resequencing reveals toll-like receptor variants contribute to human susceptibility to tuberculosis disease. *PloS one*, **2**, e1318.

127.	Uciechowski, P., Imhoff, H., Lange, C.*, et al.* (2011) Susceptibility to tuberculosis is associated with TLR1 polymorphisms resulting in a lack of TLR1 cell surface expression. *Journal of leukocyte biology*, **90**, 377-388.

128.	Ben-Ali, M., Barbouche, M.R., Bousnina, S.*, et al.* (2004) Toll-like receptor 2 Arg677Trp polymorphism is associated with susceptibility to tuberculosis in Tunisian patients. *Clinical and diagnostic laboratory immunology*, **11**, 625-626.

129.	Chen Yc Fau - Hsiao, C.-C., Hsiao Cc Fau - Chen, C.-J., Chen Cj Fau - Chin, C.-H.*, et al.* (2010) Toll-like receptor 2 gene polymorphisms, pulmonary tuberculosis, and natural. *BMC medical genetics*, **11**, 17 LID - 10.1186/1471-2350-1111-1117 [doi].

130.	Ogus Ac Fau - Yoldas, B., Yoldas B Fau - Ozdemir, T., Ozdemir T Fau - Uguz, A.*, et al.* (2004) The Arg753GLn polymorphism of the human toll-like receptor 2 gene in tuberculosis. *Eur Respir J*, **23**, 219-223.

131.	Thuong Nt Fau - Hawn, T.R., Hawn Tr Fau - Thwaites, G.E., Thwaites Ge Fau - Chau, T.T.H.*, et al.* (2007) A polymorphism in human TLR2 is associated with increased susceptibility to. *Genes Immun*, **8**, 422-428.

132.	Velez Dr Fau - Hulme, W.F., Hulme Wf Fau - Myers, J.L., Myers Jl Fau - Weinberg, J.B.*, et al.* (2009) NOS2A, TLR4, and IFNGR1 interactions influence pulmonary tuberculosis. *Human genetics*, **126**, 643-653 LID - 610.1007/s00439-00009-00713-y [doi].

133.	Ferwerda, B., Kibiki, G.S., Netea, M.G.*, et al.* (2007) The toll-like receptor 4 Asp299Gly variant and tuberculosis susceptibility in HIV-infected patients in Tanzania. *Aids*, **21**, 1375-1377.

134.	Motsinger-Reif Aa Fau - Antas, P.R.Z., Antas Pr Fau - Oki, N.O., Oki No Fau - Levy, S.*, et al.* (2010) Polymorphisms in IL-1beta, vitamin D receptor Fok1, and Toll-like receptor 2 are. *BMC medical genetics*, **11**, 37 LID - 10.1186/1471-2350-1111-1137 [doi].

135.	Najmi, N., Kaur, G., Sharma, S.K.*, et al.* (2010) Human Toll-like receptor 4 polymorphisms TLR4 Asp299Gly and Thr399Ile influence susceptibility and severity of pulmonary tuberculosis in the Asian Indian population. *Tissue antigens*, **76**, 102-109.

136.	Pulido, I., Leal, M., Genebat, M.*, et al.* (2010) The TLR4 ASP299GLY polymorphism is a risk factor for active tuberculosis in Caucasian HIV-infected patients. *Current HIV research*, **8**, 253-258.

137.	Dalgic, N., Tekin, D., Kayaalti, Z.*, et al.* (2011) Relationship between toll-like receptor 8 gene polymorphisms and pediatric pulmonary tuberculosis. *Dis Markers*, **31**, 33-38.

138.	Davila S Fau - Hibberd, M.L., Hibberd Ml Fau - Hari Dass, R., Hari Dass R Fau - Wong, H.E.E.*, et al.* (2008) Genetic association and expression studies indicate a role of toll-like receptor. *PLoS Genet*, **4**, e1000218 LID - 1000210.1001371/journal.pgen.1000218 [doi].

139.	Velez, D.R., Wejse, C., Stryjewski, M.E.*, et al.* (2010) Variants in toll-like receptors 2 and 9 influence susceptibility to pulmonary tuberculosis in Caucasians, African-Americans, and West Africans. *Human genetics*, **127**, 65-73.

140.	Correa Pa Fau - Gomez, L.M., Gomez Lm Fau - Anaya, J.M., Anaya, J.M. (2004) [Polymorphism of TNF-alpha in autoimmunity and tuberculosis]. *Biomedica*, **24 Supp 1**, 43-51.

141.	Fan Hm Fau - Wang, Z., Wang Z Fau - Feng, F.-M., Feng Fm Fau - Zhang, K.-L.*, et al.* (2010) Association of TNF-alpha-238G/A and 308 G/A gene polymorphisms with pulmonary. *Biomed Environ Sci*, **23**, 137-145 LID - 110.1016/S0895-3988(1010)60043-60048 [doi].

142.	Ma Mj Fau - Xie, L.-p., Xie Lp Fau - Wu, S.-c., Wu Sc Fau - Tang, F.*, et al.* (2010) Toll-like receptors, tumor necrosis factor-alpha, and interleukin-10 gene. *Hum Immunol*, **71**, 1005-1010 LID - 1010.1016/j.humimm.2010.1007.1009 [doi].

143.	Merza M Fau - Farnia, P., Farnia P Fau - Anoosheh, S., Anoosheh S Fau - Varahram, M.*, et al.* (2009) The NRAMPI, VDR and TNF-alpha gene polymorphisms in Iranian tuberculosis. *The Brazilian journal of infectious diseases : an official publication of the Brazilian Society of Infectious Diseases*, **13**, 252-256 LID - S1413-86702009000400002 [pii].

144.	Kusuhara K Fau - Yamamoto, K., Yamamoto K Fau - Okada, K., Okada K Fau - Mizuno, Y.*, et al.* (2007) Association of IL12RB1 polymorphisms with susceptibility to and severity of. *Int J Immunogenet*, **34**, 35-44.

145.	Mokrousov I Fau - Wu, X.-R., Wu Xr Fau - Vyazovaya, A., Vyazovaya A Fau - Feng, W.-X.*, et al.* (2011) Polymorphism of 3'UTR region of TNFR2 coding gene and its role in clinical. *Infect Genet Evol*, **11**, 1312-1318 LID - 1310.1016/j.meegid.2011.1304.1025 [doi].

146.	Shah, J.A., Vary, J.C., Chau, T.T.*, et al.* (2012) Human TOLLIP regulates TLR2 and TLR4 signaling and its polymorphisms are associated with susceptibility to tuberculosis. *Journal of immunology*, **189**, 1737-1746.

147.	Alagarasu, K., Selvaraj, P., Swaminathan, S.*, et al.* (2009) 5' regulatory and 3' untranslated region polymorphisms of vitamin D receptor gene in south Indian HIV and HIV-TB patients. *J Clin Immunol*, **29**, 196-204.

148.	Ates O Fau - Dolek, B., Dolek B Fau - Dalyan, L., Dalyan L Fau - Musellim, B.*, et al.* (2011) The association between BsmI variant of vitamin D receptor gene and. *Mol Biol Rep*, **38**, 2633-2636 LID - 2610.1007/s11033-11010-10404-11038 [doi].

149.	Banoei Mm Fau - Mirsaeidi, M.S., Mirsaeidi Ms Fau - Houshmand, M., Houshmand M Fau - Tabarsi, P.*, et al.* (2010) Vitamin D receptor homozygote mutant tt and bb are associated with susceptibility. *Int J Infect Dis*, **14**, e84-85 LID - 10.1016/j.ijid.2009.1005.1001 [doi] FAU - Banoei, Mohammad Mehdi.

150.	Chen Xr Fau - Feng, Y.-L., Feng Yl Fau - Ma, Y., Ma Y Fau - Zhang, Z.-D.*, et al.* (2006) [Study on the association of two polymorphisms of the vitamin D receptor (VDR). *Sichuan Da Xue Xue Bao Yi Xue Ban*, **37**, 847-851.

151.	Motsinger-Reif, A.A., Antas, P.R., Oki, N.O.*, et al.* (2010) Polymorphisms in IL-1beta, vitamin D receptor Fok1, and Toll-like receptor 2 are associated with extrapulmonary tuberculosis. *BMC medical genetics*, **11**, 37.

152.	Olesen R Fau - Wejse, C., Wejse C Fau - Velez, D.R., Velez Dr Fau - Bisseye, C.*, et al.* (2007) DC-SIGN (CD209), pentraxin 3 and vitamin D receptor gene variants associate with. *Genes Immun*, **8**, 456-467.

153.	Zhang, H.Q., Deng, A., Guo, C.F.*, et al.* (2010) Association between FokI polymorphism in vitamin D receptor gene and susceptibility to spinal tuberculosis in Chinese Han population. *Archives of medical research*, **41**, 46-49.

154.	Bornman L Fau - Campbell, S.J., Campbell Sj Fau - Fielding, K., Fielding K Fau - Bah, B.*, et al.* (2004) Vitamin D receptor polymorphisms and susceptibility to tuberculosis in West. *The Journal of infectious diseases*, **190**, 1631-1641.
