# Supplementary material for: HGV&TB: a comprehensive online resource on human genes and genetic variants associated with tuberculosis
Source: Database (Oxford). 2014 Dec 13;2014:bau112. doi: 10.1093/database/bau112 (PMC5630898; doi:10.1093/database/bau112)
Supplement: Supplementary Data [file bau112_Supplementary_Data.zip › Supplementary_Table_6.doc]

**Supplementary Table 6: Population frequency distribution of TB susceptible genes present in HGV&TB**

| **S. No** | **Population** | **No of variants** |
| --- | --- | --- |
| 1. | China | 74 |
| 2. | Indian | 69 |
| 3. | African- American | 44 |
| 4. | South Africa | 28 |
| 5. | Indonesia | 26 |
| 6. | Vietnam | 21 |
| 7. | West Africa (including Ghana, The Gambia, Guinea, Guinea- Bissau) | 21 |
| 8. | Japanese (including Korean) | 15 |
| 9. | Iran | 15 |
| 10. | Russia | 12 |
| 11. | Uganda | 11 |
| 12. | Mexico | 10 |
| 13. | Tunisia | 10 |
| 14. | Poland | 8 |
| 15. | Mixed | 8 |
| 16. | Turkey | 7 |
| 17. | Morocco | 7 |
| 18. | Brazil | 6 |
| 19. | Colombia | 6 |
| 20. | Spain | 5 |
| 21. | Peru | 5 |
| 22. | Malawi | 4 |
| 23. | Ethiopia | 4 |
| 24. | Caucasian | 4 |
| 25. | Cambodia | 3 |
| 26. | Zimbabwe | 2 |
| 27. | Pakistan | 2 |
| 28. | Taiwan | 2 |
| 29. | Tanzania | 2 |
| 30. | South East Asia | 1 |
| 31. | Zambia | 1 |
| 32. | Portugal | 1 |
| 33. | Italy | 1 |
