# Supplementary material for: HGV&TB: a comprehensive online resource on human genes and genetic variants associated with tuberculosis
Source: Database (Oxford). 2014 Dec 13;2014:bau112. doi: 10.1093/database/bau112 (PMC5630898; doi:10.1093/database/bau112)
Supplement: Supplementary Data [file bau112_Supplementary_Data.zip › Supplementary_Table_2.doc]

**Supplementary Table 2: List of all the Tuberculosis vulnerable gene variants (whose rsid’s are not available), having both individual and haplotype/diplotype/interaction association with Tuberculosis susceptibility uploaded in HGV&TB.**

| **S. No.** | **Genes** | **Variants** | **Reference** |
| --- | --- | --- | --- |
| 1. | IL8 | - 251T/A | Ma et. al., 2003 |
| 2. | LTA | +368G/A | Taype CA et. al. 2010 |
| 3. | TLR4 | 1196 C/T | Najmi N et. al. 2010 |
| 4. | SFTPA2 | 1382 C/G; 1492 T/C | Madan T et. al. 2002 |
| 5. | SFTPA1 | 1416 C/T; 776C/T | Madan T et. al.; .Malik S et. al., 2005 |
| 6. | CD14 | -159C/T; _ | Kang YA et. al. 2009; Rosas-Taraco AG et. al.; Zhao MY et. al. 2012 |
| 7. | P2RX7 | 1729T/A | Sambasivan V et. al.,2010 |
| 8. | IL12RB1 | -2 C/T; -111A/T | Remus N et. al. |
| 9. | TLR2 | 2029 C/T | Clin Diagn Lab Immunol. 2004 May;11 |
| 10. | CISH | 292; Position + 1320; Position + 3415 | Chiea C. Khor, M.B., B.S., D.Phil.et. al. |
| 11. | TNF | -863 A/C | Ma MJ et. al. |
| 12. | TNFRSF1A | -863 A/C | Ma MJ et. al. |
| 13. | TNFRSF1B | -863 A/C | Ma MJ et. al. |
| 14. | HLA-C | C*1200 | Louie LG et. al. |
| 15. | VDR | c.1174+283G>A (rs1544410), c.152T>C, c.152T>G, c.152T>A (rs2228570) | Sharma PR et. al. |
| 16. | MRC1 | C1303T; C1323T | Xing Z et. al. 2012 |
| 17. | HLA-DPB1 | DRB1*1502-DPB1*0201 | Selvaraj P et. al.,2008 |
| 18. | HLA-DQB1 | DRB1*1502-DQB1*0601-DPB1*0201; DQB1*0503; HLA DQB1*0601; DQB1*0601; HLA-DQB1*05; DQB1*0501; DQB1*0502; DRB1*16–DQB1*05; DRB1*04–DQB1*03; DRB1*14–DQB1*05 | Selvaraj P et. al.,2001; Goldfeld AE et. al.; Ravikumar M et. al.; Kim HS et. al.; Figueiredo JF; Terán-Escandón D et. al.; Dubaniewicz A et. al. |
| 19. | CR1 | G3093T | Jodene fitness et. al., 2004 |
| 20. | TAP1 | GG genotype at TAP1 position 333; GA genotype at TAP1 position 637 | Sunder SR et. al. |
| 21. | HLA-DQA1 | HLA DQA1*0101; HLA-DQA1*0101 | Terán-Escandón D et. al.; Amirzargar AA et. al. |
| 22. | HLA-A | HLA-A31; HLA-A*3601; HLA-A2-DRB1*1502 | Figueiredo JF; Louie LG et. al.; Raghavan S et. al. |
| 23. | HLA-B | HLA-B41; HLA-B*40:06; HLA-B40-DRB1*04; HLA-B40-DRB1*15:02; HLA-B40-DRB1*15:01 | Figueiredo JF; Raghavan S et. al. |
| 24. | HLA-DRB1 | HLA-DRB1*10; DRB1*1502-DPB1*0201; HLA DRB1*1501; HLA-DRB1*0803; DRB1*1501; HLA-DRB1*14; HLA-DRB1*07; DRB1*1502 (HLA-DRB1*15:02); HLA-A2-DRB1*1502; HLA-B40-DRB1*04; HLA-B40-DRB1*15:02; HLA-B40-DRB1*15:01; DRB1*16:01; DRB1*16–DQB1*05; DRB1*04–DQB1*03; DRB1*14–DQB1*05 | Figueiredo JF; Selvaraj P et. al.,2001; Ravikumar M et. al.; Kim HS et. al.; Terán-Escandón D et. al.; Duarte R et. al.; Amirzargar AA et. al.; Raghavan S et. al.; Dubaniewicz A et. al. |
| 25. | IL1R1 | PstI 1970 C/C | Amirzargar AA et. al., 2006 |
| 26. | CXCL12 | SDF1-A 3' UTR G/A | alagarasu et. al.,2008 |
| 27. | SP110 | sp110int10 | Tosh K et. al. 2005 |
| 28. | IL2 | T-330G | Naslednikova IO et. al. |
| 29. | IL23R | T-330G | Naslednikova IO et. al. |
| 30. | MBL2 | YB/LYD Haplotype [?550 (H/L polymorphism), ?221 (X/Y polymorphism), c.154C>T (rs5030737)]; ?221 (X/Y polymorphism) | Capparelli R et. al.; Alagarasu K, et. al. 2007 |

References

1.	Ma, X., Reich, R.A., Wright, J.A.*, et al.* (2003) Association between interleukin-8 gene alleles and human susceptibility to tuberculosis disease. *The Journal of infectious diseases*, **188**, 349-355.

2.	Taype, C.A., Shamsuzzaman, S., Accinelli, R.A.*, et al.* (2010) Genetic susceptibility to different clinical forms of tuberculosis in the Peruvian population. *Infect Genet Evol*, **10**, 495-504.

3.	Najmi, N., Kaur, G., Sharma, S.K.*, et al.* (2010) Human Toll-like receptor 4 polymorphisms TLR4 Asp299Gly and Thr399Ile influence susceptibility and severity of pulmonary tuberculosis in the Asian Indian population. *Tissue antigens*, **76**, 102-109.

4.	Madan T Fau - Saxena, S., Saxena S Fau - Murthy, K.J.R., Murthy Kj Fau - Muralidhar, K.*, et al.* (2002) Association of polymorphisms in the collagen region of human SP-A1 and SP-A2. *Clin Chem Lab Med*, **40**, 1002-1008.

5.	Malik S Fau - Abel, L., Abel L Fau - Tooker, H., Tooker H Fau - Poon, A.*, et al.* (2005) Alleles of the NRAMP1 gene are risk factors for pediatric tuberculosis disease. *Proc Natl Acad Sci U S A*, **102**, 12183-12188.

6.	Kang, Y.A., Lee, H.W., Kim, Y.W.*, et al.* (2009) Association between the -159C/T CD14 gene polymorphism and tuberculosis in a Korean population. *FEMS immunology and medical microbiology*, **57**, 229-235.

7.	Rosas-Taraco, A.G., Revol, A., Salinas-Carmona, M.C.*, et al.* (2007) CD14 C(-159)T polymorphism is a risk factor for development of pulmonary tuberculosis. *The Journal of infectious diseases*, **196**, 1698-1706.

8.	Zhang, X., Jiang, F., Wei, L.*, et al.* (2012) Polymorphic allele of human MRC1 confer protection against tuberculosis in a Chinese population. *International journal of biological sciences*, **8**, 375-382.

9.	Sambasivan V Fau - Murthy, K.J.R., Murthy Kj Fau - Reddy, R., Reddy R Fau - Vijayalakshimi, V.*, et al.* (2010) P2X7 gene polymorphisms and risk assessment for pulmonary tuberculosis in Asian. *Dis Markers*, **28**, 43-48 LID - 10.3233/DMA-2010-0682 [doi].

10.	Remus, N., El Baghdadi, J., Fieschi, C.*, et al.* (2004) Association of IL12RB1 polymorphisms with pulmonary tuberculosis in adults in Morocco. *The Journal of infectious diseases*, **190**, 580-587.

11.	Khor, C.C., Vannberg, F.O., Chapman, S.J.*, et al.* (2010) CISH and susceptibility to infectious diseases. *The New England journal of medicine*, **362**, 2092-2101.

12.	Ma Mj Fau - Xie, L.-p., Xie Lp Fau - Wu, S.-c., Wu Sc Fau - Tang, F.*, et al.* (2010) Toll-like receptors, tumor necrosis factor-alpha, and interleukin-10 gene. *Hum Immunol*, **71**, 1005-1010 LID - 1010.1016/j.humimm.2010.1007.1009 [doi].

13.	Louie, L.G., Hartogensis, W.E., Jackman, R.P.*, et al.* (2004) Mycobacterium tuberculosis/HIV-1 coinfection and disease: role of human leukocyte antigen variation. *The Journal of infectious diseases*, **189**, 1084-1090.

14.	Sharma Pr Fau - Singh, S., Singh S Fau - Jena, M., Jena M Fau - Mishra, G.*, et al.* (2011) Coding and non-coding polymorphisms in VDR gene and susceptibility to pulmonary. *Infect Genet Evol*, **11**, 1456-1461 LID - 1410.1016/j.meegid.2011.1405.1019 [doi].

15.	Selvaraj, P., Raghavan, S., Swaminathan, S.*, et al.* (2008) HLA-DQB1 and -DPB1 allele profile in HIV infected patients with and without pulmonary tuberculosis of south India. *Infection, genetics and evolution : journal of molecular epidemiology and evolutionary genetics in infectious diseases*, **8**, 664-671.

16.	Dubaniewicz, A., Moszkowska, G., Szczerkowska, Z. (2005) Frequency of DRB1-DQB1 two-locus haplotypes in tuberculosis: preliminary report. *Tuberculosis*, **85**, 259-267.

17.	Figueiredo, J.F., Rodrigues Mde, L., Deghaide, N.H.*, et al.* (2008) HLA profile in patients with AIDS and tuberculosis. *The Brazilian journal of infectious diseases : an official publication of the Brazilian Society of Infectious Diseases*, **12**, 278-280.

18.	Goldfeld, A.E., Delgado, J.C., Thim, S.*, et al.* (1998) Association of an HLA-DQ allele with clinical tuberculosis. *JAMA : the journal of the American Medical Association*, **279**, 226-228.

19.	Kim, H.S., Park, M.H., Song, E.Y.*, et al.* (2005) Association of HLA-DR and HLA-DQ genes with susceptibility to pulmonary tuberculosis in Koreans: preliminary evidence of associations with drug resistance, disease severity, and disease recurrence. *Hum Immunol*, **66**, 1074-1081.

20.	Ravikumar, M., Dheenadhayalan, V., Rajaram, K.*, et al.* (1999) Associations of HLA-DRB1, DQB1 and DPB1 alleles with pulmonary tuberculosis in south India. *Tubercle and lung disease : the official journal of the International Union against Tuberculosis and Lung Disease*, **79**, 309-317.

21.	Teran-Escandon, D., Teran-Ortiz, L., Camarena-Olvera, A.*, et al.* (1999) Human leukocyte antigen-associated susceptibility to pulmonary tuberculosis: molecular analysis of class II alleles by DNA amplification and oligonucleotide hybridization in Mexican patients. *Chest*, **115**, 428-433.

22.	Fitness, J., Floyd, S., Warndorff, D.K.*, et al.* (2004) Large-scale candidate gene study of tuberculosis susceptibility in the Karonga district of northern Malawi. *The American journal of tropical medicine and hygiene*, **71**, 341-349.

23.	Sunder, S.R., Hanumanth, S.R., Gaddam, S.*, et al.* (2011) Association of TAP 1 and 2 gene polymorphisms with human immunodeficiency virus-tuberculosis co-infection. *Hum Immunol*, **72**, 908-911.

24.	Amirzargar Aa Fau - Rezaei, N., Rezaei N Fau - Jabbari, H., Jabbari H Fau - Danesh, A.-A.*, et al.* (2006) Cytokine single nucleotide polymorphisms in Iranian patients with pulmonary. *Eur Cytokine Netw*, **17**, 84-89.

25.	Raghavan, S., Selvaraj, P., Swaminathan, S.*, et al.* (2009) Haplotype analysis of HLA-A, -B antigens and -DRB1 alleles in south Indian HIV-1-infected patients with and without pulmonary tuberculosis. *Int J Immunogenet*, **36**, 129-133.

26.	Duarte, R., Carvalho, C., Pereira, C.*, et al.* (2011) HLA class II alleles as markers of tuberculosis susceptibility and resistance. *Revista portuguesa de pneumologia*, **17**, 15-19.

27.	Selvaraj P Fau - Alagarasu, K., Alagarasu K Fau - Harishankar, M., Harishankar M Fau - Vidyarani, M.*, et al.* (2008) Regulatory region polymorphisms of vitamin D receptor gene in pulmonary. *Int J Immunogenet*, **35**, 251-254 LID - 210.1111/j.1744-1313X.2008.00764.x [doi].

28.	Tosh K Fau - Campbell, S.J., Campbell Sj Fau - Fielding, K., Fielding K Fau - Sillah, J.*, et al.* (2006) Variants in the SP110 gene are associated with genetic susceptibility to. *Proc Natl Acad Sci U S A*, **103**, 10364-10368.

29.	Naslednikova Io Fau - Urazova, O.I., Urazova Oi Fau - Voronkova, O.V., Voronkova Ov Fau - Strelis, A.K.*, et al.* (2009) Allelic polymorphism of cytokine genes during pulmonary tuberculosis. *Bull Exp Biol Med*, **148**, 175-180.

30.	Alagarasu, K., Selvaraj, P., Swaminathan, S.*, et al.* (2007) Mannose binding lectin gene variants and susceptibility to tuberculosis in HIV-1 infected patients of South India. *Tuberculosis*, **87**, 535-543.

31.	Capparelli, R., Iannaccone, M., Palumbo, D.*, et al.* (2009) Role played by human mannose-binding lectin polymorphisms in pulmonary tuberculosis. *The Journal of infectious diseases*, **199**, 666-672.
